# Single-Cell RNA Sequencing Reveals Immunomodulatory Effects of Stem Cell Factor and Granulocyte Colony-Stimulating Factor Treatment in the Brains of Aged APP/PS1 Mice

**DOI:** 10.3390/biom14070827

**Published:** 2024-07-10

**Authors:** Robert S. Gardner, Michele Kyle, Karen Hughes, Li-Ru Zhao

**Affiliations:** Department of Neurosurgery, State University of New York Upstate Medical University, 750 E. Adams Street, Syracuse, NY 13210, USA

**Keywords:** Alzheimer’s disease, hematopoietic growth factors, single-cell RNA sequencing, microglia, macrophages, myeloid cells, S100a8, S100a9

## Abstract

Alzheimer’s disease (AD) leads to progressive neurodegeneration and dementia. AD primarily affects older adults with neuropathological changes including amyloid-beta (Aβ) deposition, neuroinflammation, and neurodegeneration. We have previously demonstrated that systemic treatment with combined stem cell factor (SCF) and granulocyte colony-stimulating factor (G-CSF) (SCF+G-CSF) reduces the Aβ load, increases Aβ uptake by activated microglia and macrophages, reduces neuroinflammation, and restores dendrites and synapses in the brains of aged APPswe/PS1dE9 (APP/PS1) mice. However, the mechanisms underlying SCF+G-CSF-enhanced brain repair in aged APP/PS1 mice remain unclear. This study used a transcriptomic approach to identify the potential mechanisms by which SCF+G-CSF treatment modulates microglia and peripheral myeloid cells to mitigate AD pathology in the aged brain. After injections of SCF+G-CSF for 5 consecutive days, single-cell RNA sequencing was performed on CD11b^+^ cells isolated from the brains of 28-month-old APP/PS1 mice. The vast majority of cell clusters aligned with transcriptional profiles of microglia in various activation states. However, SCF+G-CSF treatment dramatically increased a cell population showing upregulation of marker genes related to peripheral myeloid cells. Flow cytometry data also revealed an SCF+G-CSF-induced increase of cerebral CD45^high^/CD11b^+^ active phagocytes. SCF+G-CSF treatment robustly increased the transcription of genes implicated in immune cell activation, including gene sets that regulate inflammatory processes and cell migration. The expression of S100a8 and S100a9 was robustly enhanced following SCF+G-CSF treatment in all CD11b^+^ cell clusters. Moreover, the topmost genes differentially expressed with SCF+G-CSF treatment were largely upregulated in S100a8/9-positive cells, suggesting a well-conserved transcriptional profile related to SCF+G-CSF treatment in resident and peripherally derived CD11b^+^ immune cells. This S100a8/9-associated transcriptional profile contained notable genes related to pro-inflammatory and anti-inflammatory responses, neuroprotection, and Aβ plaque inhibition or clearance. Altogether, this study reveals the immunomodulatory effects of SCF+G-CSF treatment in the aged brain with AD pathology, which will guide future studies to further uncover the therapeutic mechanisms.

## 1. Background

Alzheimer’s disease (AD) is the most prominent neurodegenerative disease and the leading cause of dementia [1,2]. The primary risk factor for AD is advanced age. Strikingly, approximately one in nine Americans over the age of 65 is currently living with AD-related dementia. Moreover, the societal impacts of AD are immense, with annual healthcare costs greater than $320 billion [2]. Without novel effective treatments, the number of Americans afflicted with AD is projected to reach 13.8 million by 2060 [3].

The neuropathological hallmarks of AD include extracellular plaques composed of aggregated amyloid-beta (Aβ), intracellular neurofibrillary tangles composed of aggregated tau, and neuroinflammation [4,5]. Aβ peptides generated by the sequential proteolysis of amyloid precursor protein (APP) by β- and γ-secretases are particularly prone to aggregation [6,7], resulting in plaques that contribute to tau pathology, neuroinflammation, synaptic dysfunction, neurodegeneration, and cognitive decline [8,9,10,11,12,13].

Several studies crossing Aβ and tau transgenic animal models that develop extracellular plaques and intracellular tangles, respectively, revealed that while Aβ pathology enhanced the formation of neurofibrillary tangles, tau pathology had relatively little effect on Aβ deposits [14,15,16]. Injections of Aβ fragments, oligomers, or fibrils into the brain are shown to enhance the formation of neurofibrillary tangles [17], increase neuroinflammation and neurodegeneration, and impair spatial learning and memory [18,19,20]. These collective findings suggest that Aβ neuropathology is a contributory factor and a relatively early event in the progression of AD, and that Aβ clearance is a promising focus for therapeutic advancements [21]. Indeed, the clearance of Aβ from the brain is associated with the mitigation of neurodegeneration and cognition decline, largely observed in mouse models [22,23,24].

Effective and safe treatments that can stop or delay the pathological progression for AD patients, however, are not currently available. The approved and prescribed treatments for AD were primarily symptomatic, for example, targeting behavioral problems rather than the underlying neuropathology, and thus, do not stop or reverse the progression of the disease [25]. Anti-Aβ monoclonal antibodies have been approved by the United States Food and Drug Administration for the treatment of early AD patients presenting with Aβ pathology, and they have been shown to reduce Aβ and improve cognitive function; however, concerns over Aβ antibody treatments include high treatment costs, accessibility, and the incidence of adverse events including brain edema, microhemorrhages, and brain volume loss [26,27,28]. It remains highly imperative to develop safe and effective treatments for AD patients.

Microglia are the primary phagocytic cells in the brain. They act as the first line of immune defense, surveilling the microenvironment and clearing debris or pathogens, including aggregated Aβ plaques [29], to maintain homeostasis [30]. In AD, bone marrow-derived blood myeloid cells, most notably, monocytes, augment the brain’s immune cell population by migrating to the brain and differentiating into macrophages, also showing robust efficacy to uptake and degrade aggregated Aβ [31,32,33]. Notably, mutations in genes related to microglial and macrophage activation, e.g. triggering receptor expressed on myeloid cells 2 (*Trem2*), a transmembrane receptor activated by Aβ [34] and associated with Aβ clearance [35,36], confer a relatively high risk of AD [37,38].

Microglia and macrophages that migrate to and surround Aβ plaques release a host of extra-cellular vesicles and soluble molecules, including cytokines with inflammatory and/or anti-inflammatory functions, and thus, additionally regulate neuroinflammatory processes [39,40]. Activated microglia and macrophages associated with a relatively anti-inflammatory profile are thought to confer debris clearance without significantly contributing to inflammation and may enhance angiogenesis and tissue repair [41,42,43]. However, during the progression of AD pathology, microglia and macrophages increasingly take on a relatively pro-inflammatory state [44,45,46]. Although numerous reports demonstrate that Aβ clearance is enhanced by pro-inflammatory signaling or cytokines [23,47,48,49,50], persistent or chronic inflammation is generally thought to enhance Aβ spreading and aggregation [51], creating a vicious cycle leading to neurodegeneration [52]. Altogether, these previous studies strongly suggest that the development of novel AD therapies to clear Aβ from the brain should consider their effects on inflammation.

Stem cell factor (SCF) and granulocyte colony-stimulating factor (G-CSF) are two hematopoietic growth factors that synergistically stimulate the proliferation, differentiation, and mobilization of hematopoietic stem cells and progenitor cells, significantly increase the population of blood leukocytes, and augment the immune response [53,54,55,56]. SCF and G-CSF also act directly on neurons, glia, and blood vessel cells [57,58,59] in the central nervous system. SCF and G-CSF treatment enhances angiogenesis, neural survival, neurite outgrowth, synaptogenesis, and neurogenesis, and reduces neuroinflammation in several models of neurodegenerative diseases, neurological disorders, and brain trauma [60,61,62,63,64,65,66]. In AD patients, plasma levels of SCF and G-CSF are significantly decreased [67,68]. Clinical studies have also demonstrated inverse correlations between the plasma levels of SCF or G-CSF and AD severity [68,69] and Aβ levels in cerebrospinal fluid [70].

Demonstrating the efficacy of SCF and G-CSF to treat AD neuropathology in pre-clinical studies, our lab has observed that the systemic treatment of combined SCF and G-CSF (SCF+G-CSF) leads to long-lasting reductions in Aβ plaques in the hippocampus and cortex of middle-aged [71] and aged [22] APP/PS1 mice, a commonly used mouse model of Aβ pathology in AD research. Coincident with these findings, SCF+G-CSF treatment increases the association between Aβ plaques and activated Trem2^+^ microglia and macrophages, increases Aβ contained in CD68^+^ lysosomal compartments, increases the density of the anti-inflammatory marker IL-4, and decreases the density of the pro-inflammatory marker NOS-2 in the hippocampus and cortex of aged APP/PS1 mice [22]. These collective findings suggest that SCF+G-CSF treatment enhances Aβ clearance by activating microglia and macrophages to uptake and degrade Aβ plaques, while also shifting the environment toward a relatively anti-inflammatory state. Importantly, these effects further correspond to SCF+G-CSF-mediated reductions in aggregated tau and increases in the dendritic marker MAP2 and post-synaptic marker PSD-95 in the brains of aged APP/PS1 mice, suggesting the treatment-related rebuilding of neural connections [22]. Thus, SCF+G-CSF treatment in aged APP/PS1 mice ameliorates or reverses each central feature of AD neuropathology: aggregated Aβ, Aβ-induced aggregation of tau, neuroinflammation, and the degeneration of neural processes and synaptic connections [22]. These findings indicate that SCF+G-CSF treatment changes the functions of microglia and macrophages to mitigate AD neuropathology in the aged brain.

Leveraging a single-cell RNA sequencing approach, the aim of the present study is to identify novel transcriptional profiles of microglia and myeloid cells in the brains of aged APP/PS1 mice following SCF+G-CSF treatment. Similar approaches have been used to identify novel functional states of microglia related to AD, including a profile of disease-associated microglia (DAM) found to limit the progression of AD pathology [72,73]. Here, we analyzed the transcriptional profiles of CD11b^+^ microglia and myeloid cells isolated from the brains of aged APP/PS1 mice treated with SCF+G-CSF or vehicle solutions. We profiled the transcriptional changes of brain CD11b^+^ cells the day after a 5-day treatment period. The findings of this study provide the unbiased identification of SCF+G-CSF treatment-related transcriptional profiles of immune cells in the brain, which may guide future mechanistic studies to understand how SCF+G-CSF treatment mitigates AD pathology.

## 2. Materials and Methods

### 2.1. Animals

All methods were carried out in accordance with the National Institutes of Health Guide for the Care and Use of Laboratory Animals and approved by the State University of New York Upstate Medical University Institutional Animal Care and Use Committee. Aged APP/PS1 mice (male, 28 months old) (stock# 034832, Jackson Laboratory, Bar Harbor, ME, USA) were used in these experiments. APP/PS1 mice express both chimeric amyloid precursor protein (human APP695swe) with Swedish double mutations (K595N/M596L) and human presenilin protein 1 carrying the exon-9-deleted variant (PS1-dE9) [74]. APP/PS1 mice develop plaques of human amyloid-beta (Aβ) peptide in the brain by 6–7 months of age. Mice had free access to food and water and were housed in a 12 h light/dark cycle. The health status of the mice was checked daily. 

### 2.2. Experimental Design

**Figure 1:**
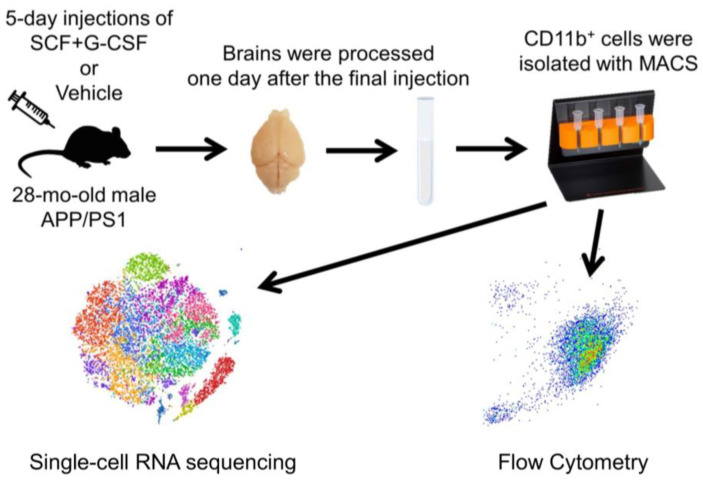
Experimental flow chart. APP/PS1 mice (28-month-old; male) were subcutaneously injected with either combined stem cell factor (SCF) and granulocyte colony-stimulating factor (G-CSF) or vehicle solution for 5 days. The next day, whole brains were excised and processed into single-cell suspensions. After debris and dead cells were removed, CD11b^+^ cells were isolated using magnetic-activated cell sorting (MACS). The isolated CD11b^+^ cells were used for either single-cell RNA sequencing or flow cytometry.

The experimental design is summarized in Figure 1. Twelve APP/PS1 mice at the age of 28 months old were subcutaneously injected with stem cell factor (SCF, PeproTech, Rocky Hill, NJ, USA; 200 µg/kg in saline) and granulocyte colony-stimulating factor (G-CSF, Amgen, Thousand Oaks, CA, USA; 50 µg/kg in 5% dextrose) or an equal volume of vehicle solution (n = 6 in each group) for 5 consecutive days. On the morning of day 6 after completing the 5-day injections, whole brains (3 mice in each group) were removed and dissociated. CD11b^+^ cells were isolated using magnetic-activated cell sorting (MACS). The isolated CD11b^+^ cells from the brains of 3 mice in the same treatment group were pooled together for single-cell RNA sequencing (scRNAseq). The isolated CD11b^+^ cells were also analyzed using flow cytometry for immuno-phenotyping. Flow cytometry was performed on aliquots of the pooled CD11b^+^ cells used for scRNAseq and on CD11b^+^ cells of individual samples isolated from the brains of 6 additional mice (3 in each group).

### 2.3. Cell Processing and CD11b^+^ Isolation for Single-Cell RNA Sequencing and Flow Cytometry

*Cell dissociation.* Mice were anesthetized with Ketamine (100 mg/kg) and Xylazine (10 mg/kg) (i.p.) and euthanized by intracardiac perfusion with ice-cold heparinized (10k U/L; NDC#25021-403-04; Sagent Pharmaceuticals, Schaumburg, IL, USA) Dulbecco’s phosphate-buffered saline (D-PBS; Cat#14190136; ThermoFisher, Liverpool, NY, USA). Each brain was removed, immersed in a cell culture dish containing ice-cold D-PBS+ (D-PBS supplemented with calcium, magnesium, glucose, and pyruvate; Cat#14287080; ThermoFisher, Liverpool, NY, USA), and minced (~1–2 mm in any dimension) using an ice-cold sterile scalpel blade. Brains were dissociated using the commercially available Adult Brain Dissociation Kit (Cat#130-107-677; Miltenyi Biotec, Gaithersburg, MD, USA) and the Gentle MACS Octo Dissociator with Heaters (Cat#130-096-427; Miltenyi Biotec, Gaithersburg, MD, USA) according to manufacturer specifications. Briefly, tissue pieces from each brain were transferred into a C-tube (Cat#130-096-334; Miltenyi Biotec, Gaithersburg, MD, USA) containing 1950 µL of enzyme mix 1. Enzyme mix 2 was subsequently added (30 µL), and the sample was dissociated using the program 37C_ABDK_01. Each sample was spun down, and the re-suspended pellet was passed through pre-wet 400 µM (Cat#43-50400-03; PluriSelect, El Cajon, CA, USA) and 70 µM cell filters (Cat#43-50070-51; PluriSelect, El Cajon, CA, USA). Each cell suspension was centrifuged at 300× *g* for 10 min at 4 °C, and the cell pellet was re-suspended in 3.1 mL D-PBS+ for subsequent processing to clear debris and dead cells.

*Debris and dead cell removal.* Debris removal solution (900 µL; Cat#130-109-398; Miltenyi Biotec, Gaithersburg, MD, USA) was mixed into each suspension and 4 mL of D-PBS+ was layered over top. The samples were centrifuged at 3000× *g* for 10 min at 4 °C. The top and debris inter-phases were removed, and the remaining solution was diluted in D-PBS+ (14 mL), inverted three times, and centrifuged at 1000× *g* for 10 min at 4 °C. Dead and dying cells were removed using the Dead Cell Removal kit from Miltenyi Biotec (Cat#130-090-101, Gaithersburg, MD, USA). Briefly, the pellets were resuspended and incubated for 15 min at room temperature in 100 µL of the dead cell removal magnetically labeled antibody. Binding Buffer (400 µL) was added to the suspension, which was subsequently passed through a pre-washed LS column (Cat#130-042-401; Miltenyi Biotec, Gaithersburg, MD, USA; 1 LS column per brain), placed in a strong magnetic field (Quadro MACS Separator, Cat#130-090-976, attached to the MACS MultiStand, Cat#130-042-303; Miltenyi Biotec, Gaithersburg, MD, USA), and topped with a pre-wet 70 µM cell filter. Each filter/column was further washed 4 times with Binding Buffer. Flow-through, largely depleted of dead cells (confirmed by trypan blue staining of cell aliquots), was collected and centrifuged at 300× *g* for 10 min at 4 °C. Each pellet was resuspended in 1 mL 1xMACS buffer (diluted in PBS; Cat#130-091-376; Miltenyi Biotec, Gaithersburg, MD, USA) and passed through a 40 µM cell filter (Cat#43-50040-51; PluriSelect, El Cajon, CA, USA). Additional MACS buffer (4 mL) was passed through the 40 µM filter to maximize cell recovery. The suspension from each brain was centrifuged at 300× *g* for 10 min at 4 °C, and each pellet was resuspended in 270 µL MACS buffer prior to magnetic activated cell sorting of CD11b^+^ brain cells. 

*Magnetic-activated cell sorting of CD11b^+^ brain cells.* CD11b ultra-pure micro beads (30 µL; Cat#130-126-725; Miltenyi Biotec, Gaithersburg, MD, USA) were incubated with the sample for 15 min at 4 °C. Subsequently, each cell suspension was washed with 2 mL MACS buffer and centrifuged at 300× *g* for 10 min at 4 °C. Each pellet was resuspended in 500 µL MACS buffer and passed through a pre-washed LS column (1 LS column per brain) placed in the magnetic field. Each LS column was topped with a pre-wet 70 µM cell filter. Each filter/column was further washed 3 times with MACS buffer. To enhance CD11b purity, the magnetically captured cells were eluted into a second LS column affixed to the magnet. The second LS column was washed 3 times with MACS buffer. Viability of magnetically captured cells (eluted into a new tube away from the magnet) was assessed by trypan blue staining (1:2 dilution sample in trypan blue; Cat#1450013; Bio Rad, Hercules, CA USA). Cell viability was quantified as the proportion of cells that did not take up the dye. Aliquots were further processed for scRNAseq and for flow cytometry. 

### 2.4. Single-Cell RNA Sequencing

The cell suspensions were centrifuged at 300× *g* for 10 min at 4 °C, and the pellets were resuspended in DMEM/F12 (Cat#11320033; ThermoFisher, Liverpool, NY, USA) supplemented with Fetal Bovine Serum (10%) to achieve a concentration of 1000 cells/µL. The cell solutions were placed on ice and run for scRNAseq using the Chromium single cell gene expression platform (10x Genomics, Pleasanton, CA, USA) to target 10,000 cells. Following manufacturer specifications (GEM Single Cell 3′ Reagent Kit v3.1: 10x Genomics, Pleasanton, CA, USA), samples and barcoded gel beads were loaded onto a G chip and run on a Chromium Controller to partition single cells for generation of cell-specific barcoded cDNA. Pooled cDNA was sequenced using the Illumina NextSeq 500 High Output Kit. The single-cell RNA sequencing was performed by the Molecular Analysis Core Facility at SUNY Upstate Medical University.

### 2.5. Flow Cytometry

Separate aliquots of cell suspensions used for scRNAseq were centrifuged at 300× *g* for 10 min at 4 °C, and the pellets were resuspended in D-PBS supplemented with 1% BSA. Aliquots (100 µL) were incubated with anti-mouse CD11b-APC (1:50 dilution; Cat#130-113-802; Miltenyi Biotec, Gaithersburg, MD, USA) and anti-mouse CD45-PE (1:50 dilution; Cat#130-110-797; Miltenyi Biotec, Gaithersburg, MD, USA) for 10 min at 4 °C. Separate aliquots were incubated with control isotype antibodies. Cells were washed with 2 mL D-PBS (1% BSA) and centrifuged at 300× *g* for 10 min at 4 °C. The pellets were resuspended in 600 µL D-PBS (1% BSA) for flow cytometry analysis. Additional samples were stained and analyzed as above using the following modifications: the samples were blocked with FcR block (1:200 dilution; Cat#553141; BD Biosciences, Franklin Lakes, NJ, USA) for 10 min at 4 °C and incubated with anti-mouse CD11b-PE (1:50 dilution; Cat#130-113-806, Miltenyi Biotec, Gaithersburg, MD, USA) and anti-mouse CD45-APC (1.15:100 dilution; Cat#103112; Biolegend, San Diego, CA, USA) for 15 and 30 min, respectively, at 4 °C. SCF+G-CSF-treated and vehicle-control samples were counterbalanced across all experiments. Cells were assayed on the BD LSR-II cytometer with FACSDiva software v9 (BD Biosciences, Franklin Lakes, NJ, USA) using 561 nM and 633 nM excitation lasers paired with detection filters 585/15 nM and 630/20 nM for PE and APC signals, respectively. 

### 2.6. Data Analysis and Statistics

*Single-cell RNA sequencing.* Analysis and figure generation of scRNAseq data were primarily performed using Partek Flow software (Build version 11.0.24.0624). Reads were trimmed and aligned to the mm10 assembly using Bowtie2. To ensure the results presented here were robust to choice of aligners, primary findings were corroborated using alternate aligners, including STAR. To limit artifacts, unique molecular identifiers (UMIs) that identify individual transcripts were de-duplicated. Moreover, cell-specific barcodes were filtered to limit those not associated with a cell, using default parameters. A single cell count matrix was generated by quantifying the reads for each cell barcode to mm10_refseq_v97 annotations. Based on recommendations for using 10x Genomics kits, the minimum read overlap for inclusion in the count matrix was set to 50% of read length. Strand specificity was set to Forward-Reverse. Low quality cells (e.g., doublets, those with few reads) were manually filtered out based on the distributions of read counts, detected genes, and ribosomal counts across the entire dataset. Manual filtering was done blindly with respect to treatment condition and individual gene profiles. This method yielded a total of 19,008 cells. Normalization was performed to account for differences in total UMI counts per cell and values were log2-transformed. To reduce noise and limit analysis of genes with little-to-no expression, genes with counts less than 1 in 99% of cells were excluded from analysis. Data were dimensionally reduced by principal component analysis. The 10 principal components explaining the most variance in the dataset were used to identify clusters. Unsupervised cluster analysis was performed using the Louvain algorithm. t-Distributed stochastic neighbor embedding (tSNE) dimensional reduction plots were generated to visualize cell clusters and their associated gene expression profiles.

To evaluate changes across treatment groups (SCF+G-CSF vs. Vehicle) in cell distributions across clusters, chi-square analysis was performed (Graph Pad v9) with alpha set to 0.05. Differentially expressed genes across conditions were identified using the Biomarker function in Partek. For restricted gene sets (e.g., homeostatic or DAM gene sets), differentially expressed genes showing a ≥0.4 log2-fold change across conditions with a false discovery rate (FDR)-adjusted (step-up method) *p* value < 0.05 [75] were highlighted as significant. For analyses across the entire transcriptome, significance thresholds were set to a ≥log2-fold change across conditions with an FDR-adjusted *p* value < 0.01. This threshold limited significant hits to those genes most robustly altered by SCF+G-CSF treatment. The bioinformatics tool VolcaNoseR [76] was used to generate volcano plots of differentially expressed genes. Enriched gene ontologies, Reactome pathways, and gene interaction networks (generated by querying the STRING database) were identified and analyzed using default settings in Cytoscape 3.9.1 [77,78].

**Figure 2:**
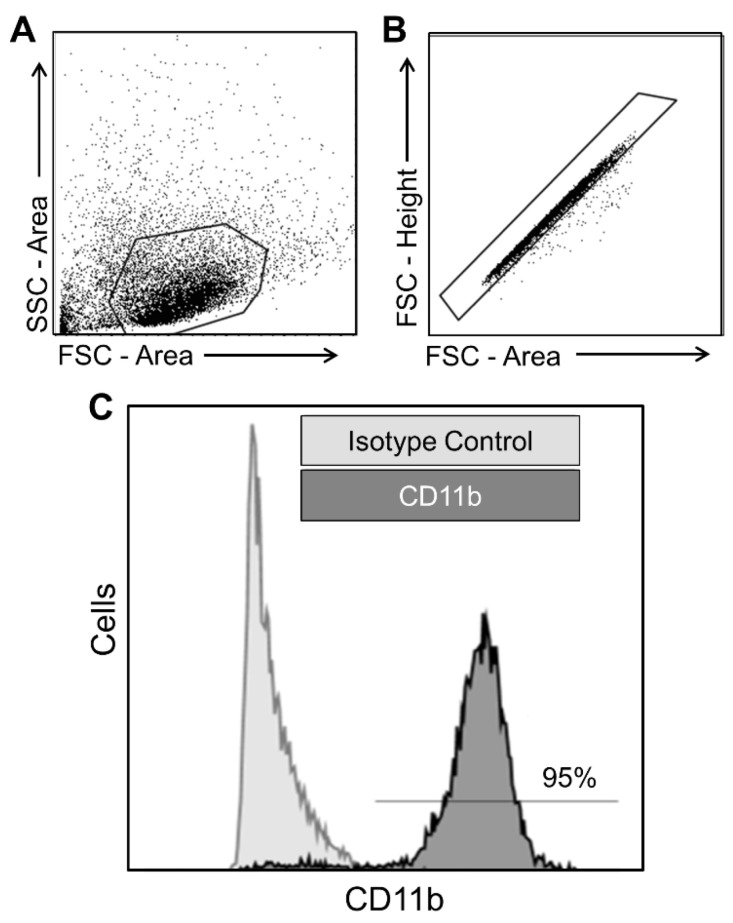
MACS-isolated CD11b^+^ cells show a high degree of purity. Gating strategies using forward scatter (FSC) and side scatter (SSC) profiles exclude (**A**) cell debris and (**B**) cell multiplets from the MACS-isolated CD11b^+^ cell suspension. (**C**) Representative data of flow cytometry. Relative to signal from isotype controls, ~95% of the single cells show expression for CD11b.

*Flow Cytometry.* Data processing was performed using Flow Jo v10. Debris was gated out using forward and side scatter profiles, and single cells were selected based on forward scatter area by forward scatter height (Figure 2A,B). Isotype-stained controls were used for thresholding and identification of CD11b^+^ and CD45^+^ cells. CD11b purity of the MACS-isolated samples was estimated as the percentage of single cells positive for CD11b. CD11b^+^/CD45^high^ populations were manually gated blind to treatment condition. Outcome measures and findings across treatment groups were robust to the use of distinct antibodies and fluorochromes. Thus, all flow cytometry data within each treatment group were combined for subsequent presentation and analysis. An independent samples t-test was performed (Graph Pad Prism v9) to compare the proportion of CD11b^+^ cells contained in the CD45^high^ gate across treatment groups with alpha set to 0.05. 

## 3. Results

### 3.1. The Isolated CD11b^+^ Cells Show a High Degree of Purity and Viability

To assess the purity of the MACS-isolated CD11b^+^ cells from the brains of aged APP/PS1 mice, flow cytometry was used to quantify the cells showing surface expression of CD11b (Figure 2). We observed that 95% of the MACS-isolated cells expressed CD11b (Figure 2C). The MACS-isolated CD11b^+^ cells also showed a high degree of viability, as assessed by trypan blue staining (95% viable). These findings indicate that our cell isolation method is effective, yielding highly pure and viable CD11b^+^ cells isolated from the brains of aged APP/PS1 mice.

### 3.2. The Vast Majority of CD11b^+^ Cells Isolated from the Brains of Aged APP/PS1 Mice Have Transcriptional Profiles That Align with Microglia and Myeloid Cells

**Figure 3:**
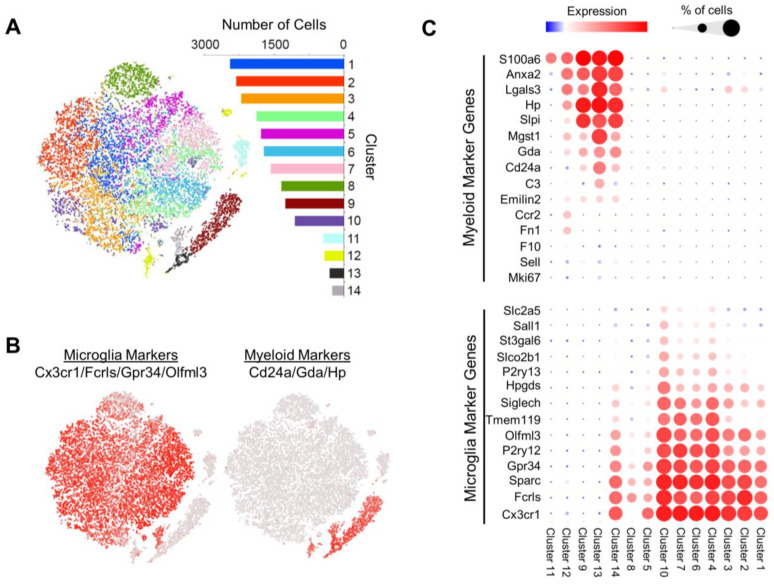
Cluster analysis of the single-cell RNA sequencing dataset reveals 14 cell clusters largely differentiated by marker genes of microglia and peripherally derived myeloid cells. (**A**) t-distributed stochastic neighbor embedding (tSNE) dimensionality reduction plots of 19,008 cells that are classified into 14 unique graph-based clusters defined and color-coded by unique transcriptional profiles. The number of cells in each cluster is shown in the corresponding bar graph. (**B**) Expression (in red) of microglial marker genes across tSNE plots indicates that the vast majority of clusters (1–8, 10, 14) associate with a microglia transcriptional signature. Expression (in red) of peripherally derived myeloid cell marker genes across tSNE plots indicates that several clusters (9, 12–14) associate with a myeloid cell transcriptional signature. Note, cluster 14 highly expresses genes associated with both cell classes. (**C**) Clusters 1–8 and 10 show enrichment of microglia gene profiles, while clusters 9, 12, and 13 show enrichment of myeloid cell profiles. Cluster 14 shows expression of marker genes of both cell types, while cluster 11 does not show expression of genes in either category. Bubble plots: the diameters of the circles correspond to the percentage of cells that express a given gene, while the color intensities of the circles correspond to the magnitude of expression.

Next, scRNAseq was performed on the MACS-isolated CD11b^+^ cells from the brains of vehicle controls and SCF+G-CSF-treated old APP/PS1 mice. Gene transcription profiles were analyzed in a total of 19,008 cells, and tSNE plots were constructed to visualize expression patterns (Figure 3). Unsupervised cluster analysis across all cells revealed 14 relatively unique clusters, which were color-coded and ranked according to cell count (Figure 3A).

The cell clusters highly expressed numerous genes commonly used to identify CD11b^+^ microglia and macrophages. *Cst3*, *Lyz2*, and *Tyrobp* showed relatively robust expression across individual clusters (Figure S1) (Supplemental Data File S1). In contrast, these clusters did not express marker genes of neurons, neuron progenitor cells, astrocytes, oligodendrocytes, oligodendrocyte progenitor cells, vascular cells, and fibroblasts (Figure S1). These results confirm the purity of the MACS-isolated CD11b^+^ cells and suggest that the isolated CD11b^+^ cells are primarily composed of microglia and peripheral myeloid cell populations.

To further characterize the primary cell classes contained in each cluster, we analyzed the expression of gene sets identified by Haage and coworkers [79] to differentiate microglia from myeloid cells thought to represent monocytes/macrophages. We found that the majority of clusters (clusters 1–8 and 10, containing 85.9% of all cells) aligned with microglia gene profiles (*Cx3cr1^+^*, *Fcrls^+^*, *Sparc^+^*, *Gpr34^+^*, *P2ry12^+^*, *Olfml3^+^*, *Tmem119^+^*, *Siglech^+^*, *Hpgds^+^*, *P2ry13^+^*, *Slco2b1^+^*, *St3gal6^+^*, *Sall1^+^*, and *Scl2a5^+^*) but not monocyte/macrophage profiles (*S100a6^+^*, *Anxa2^+^*, *Lgals3*, *Hp^+^*, *Slpl^+^*, *Mgst1^+^*, *Gda^+^*, *CD24a^+^*, *C3^+^*, *Sell^+^*, *Emilin2^+^*, *Mki67^+^*, *F10^+^*, *Fn1^+^*, and *Ccr2^+^*) (Figure 3B,C and Figure S2). In stark contrast, the gene profiles of clusters 9, 12, and 13 (containing 10.5% of cells) largely aligned with those of monocytes/macrophages but not with those of microglia (Figure 3B,C). Consistent with these results, as compared to clusters 1–8 and 10, clusters 9, 12, and 13 showed a significantly higher expression of *Lgals3* (Figure 3C and Figure S2), an additional proposed marker gene of monocytes/macrophages following their migration to the brain [80]. Cluster 14 (containing 1.3% of cells) is a unique cluster showing robust expression of both microglial and monocyte/macrophage marker genes (Figure 3C). Subsequent analysis of cluster 14 demonstrated a significant expression of *S100a6*, *Anxa2*, *Hp*, *Lgals3*, *Slpi,* and *Gda* (all monocyte/macrophage markers) in *Cx3cr1^+^*, *P2ry12^+^*, *Fcrls^+^*, *Gpr34^+^*, and *Sparc^+^* cells (Table S1), suggesting the robust co-expression of monocyte/macrophage and microglial gene sets in a small group of CD11b^+^ cells.

These collective results largely point to differentiated cell clusters of microglia and monocyte/macrophages. However, we note that given the chronic and severe neuropathological and neuroinflammatory conditions in the brains of aged APP/PS1 mice, it remains challenging to definitively distinguish microglia from peripherally derived macrophages based on transcriptional profiles alone [79,81]. Additionally, microglia and/or monocytes/macrophages express genes that are also expressed and/or used as marker genes in neutrophils [82,83,84,85], particularly under inflammatory conditions. These findings present a challenge to definitively differentiating sub-types of these cells. Considering these challenges, here, we classify cell clusters 1–8 and 10 as microglial signature (MG-sig) clusters, based on their microglial cell-like transcriptional profiles, and clusters 9, 12, and 13 as myeloid signature (Mye-sig) clusters, based on their transcriptional profiles that are similar to peripherally derived myeloid cells or monocytes/macrophages. Likewise, we classify cluster 14 as a MG/Mye-sig cluster, based on a transcriptional profile that aligned with both microglia and monocytes/macrophages.

While cluster 11 (containing 2.3% of cells) highly expressed *S100a6*, very little expression was observed of the remaining microglial and monocyte/macrophage gene markers (Figure 3C). The inspection of gene sets associated with additional immune cell classes revealed a relatively high expression of *Cd3d*, *Cd8b1*, and *Cd8a* in cluster 11 (Figure S1), demonstrating a profile consistent with that of T-cells [86].

Altogether, these findings suggest that the CD11b^+^ cells isolated from the brains of aged APP/PS1 mice have transcriptional profiles that align with those of microglia and peripheral myeloid cells, with a small population of cells showing a profile resembling that of T-cells. Given prior findings that SCF+G-CSF treatment induces changes in microglia and macrophages to engulf Aβ [22], the current study focuses strictly on the microglial cell-like MG-sig clusters and the myeloid cell-like Mye-sig clusters.

### 3.3. Microglial Signature Clusters Lie across a Gradient of Transcriptional Profiles Largely Associated with Activation and Disease States

**Figure 4:**
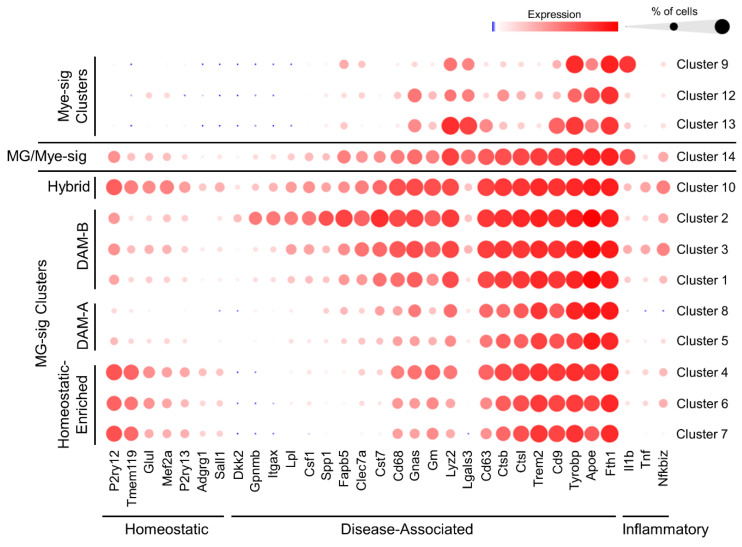
Expression profiles of homeostatic, DAM, and inflammatory gene sets reveal a gradient of activation states across clusters of CD11b^+^ cells isolated from the brains of aged APP/PS1 mice. Expression of select gene sets linked to homeostatic microglia, disease associated microglia (DAM), and inflammation, are plotted across cell clusters. Homeostatic gene markers (e.g., *Tmem119* and *P2ry12*) are highly expressed in microglia-signature clusters 4, 6, and 7, while these clusters show relatively little expression of select DAM gene markers (e.g., *Itgax*, *clec7a*, *Cst7*, *Lpl*, and *Fabp5*). These clusters were classified as MG-sig clusters with relative enrichment of homeostatic marker genes (i.e., homeostatic-enriched). In contrast, MG-sig cell clusters 1, 2, 3, 5, and 8 show high expression of DAM genes with relatively little expression of homeostatic genes. These MG-sig clusters are classified as DAM-A (clusters 5 and 8) and DAM-B (clusters 1, 2, 3) according to the numbers of expressed DAM genes and levels of expression, with DAM-B showing a higher expression of more DAM genes. MG-sig cluster 10 has a transcriptional profile relatively enriched in both homeostatic and DAM genes and is characterized as a hybrid cluster. Mye-sig clusters 9, 12, and 13, while also expressing *Tyrobp*, *Apoe*, and *Fth1*, are largely associated with selective increases in *Lgals3* and reduced expression of the majority of DAM genes. MG/Mye-sig cluster 14 shows a DAM-B profile. Bubble plots: the diameters of the circles correspond to the percentage of cells that express a given gene, while the color intensities of the circles correspond to the magnitude of expression.

Differential gene analysis across cell clusters revealed gene sets relatively unique to each cluster. Across the MG-sig clusters, many top differentially expressed genes (DEG) were associated with homeostatic (e.g., *Tmem119*, *P2ry12*) or reactive disease states (e.g., *Apoe*, *Cst7*, *Trem2*, *Itgax*, *Lpl*, and *Clec7a*) [87,88]. Emerging evidence demonstrates the role of highly reactive disease-associated microglia (DAM) [72] in slowing the progression of AD pathology [88]. Interestingly, this DAM state is associated with changes in the expression levels of many of the top marker genes we noted across our MG-sig clusters. Moreover, DAM can take on distinct inflammatory profiles that correspond to phagocytic efficacies [89]. Given the overlap of DAM gene sets and our cluster-specific marker genes, we probed expression patterns of curated gene sets linked to homeostatic and DAM profiles [87] and to inflammation across clusters. To best characterize the diversity of the MG-sig clusters (1–8, and 10), genes with robust or variable expression patterns across clusters were selected and shown in Figure 4. While all MG-sig clusters expressed high levels of select DAM signature genes, including *Trem2*, *Apoe*, and *Tyrobp*, select homeostatic and DAM marker genes were variably expressed and generally demarcated the clusters (Figure 4). Homeostatic genes (e.g., *Tmem119*, *P2ry12*, and *Glul*) were robustly enriched in clusters 4, 6, and 7, coinciding with a reduction of some DAM genes (e.g., *Lpl*, *Fabp5*, *Clec7a*, and *Cst7*). Homeostatic genes, however, were downregulated in clusters 1, 2, 3, 5, and 8. Clusters 5 and 8 showed a modest expression of lysosomal marker gene *Cd68* and increased expression of relatively few variably expressed DAM genes (e.g., *Cst7* and *Fabp5*). In contrast, clusters 1, 2, and 3 displayed a heightened expression of *Cd68* and the DAM genes *Lyz2*, *Cst7*, *Clec7a*, *Fabp5*, *Spp1*, *Lpl*, *Itgax*, *Apoe*, and *Csf1*, consistent with other reports suggesting an advanced or later-stage DAM profile [88] thought to further regulate phagocytic and lipid metabolic functions. Cluster 3 also expressed relatively high levels of *Nfkbiz*, *Tnf*, and *Il1b* genes, suggesting an inflammatory profile.

MG-sig cluster 10 showed a robust expression of the majority of DAM and homeostatic marker genes (Figure 4). This profile was confirmed in individual Tmem119^+^ and P2ry12^+^ cells (Table S2), suggesting high levels of DAM and homeostatic marker genes in a subset of MG-sig cells. This cluster additionally showed relatively high expression levels of inflammatory genes (Nfkbiz, Tnf, and Il1b). These findings reveal a population of MG-sig cells characterized by high levels of homeostatic, DAM, and inflammatory signature genes.

The transcriptional profiles across the MG-sig cells found here appear to represent a wide range of transitional states that, with the exception of a small subset (cluster 10), highlight an inverse relationship between the enrichment of homeostatic genes and the enrichment of DAM genes. For subsequent analysis, the MG-sig clusters were grouped into four distinct hubs based on their relative expression profiles of homeostatic and DAM gene sets (Figure 4): (1) an MG-sig cluster hub showing relatively high expression levels of homeostatic genes and relatively low expression levels of DAM genes (clusters 4, 6, and 7; characterized as a homeostatic-enriched hub), (2) a relatively reactive MG-sig hub with modest enrichment of DAM signature genes (clusters 5 and 8; characterized as DAM-A), (3) an MG-sig hub relatively enriched with a high degree of DAM signature genes that corresponds to an advanced DAM stage (clusters 1, 2, and 3; characterized as DAM-B), and (4) an MG-sig hub highly enriched with both homeostatic and DAM genes (cluster 10; characterized as a hybrid hub). While we observed between-cluster and intra-cluster variation, our cluster hub classifications reflect robust changes in the transcriptional profiles of cluster-specific marker genes (Figure 4). It is worth noting that all MG-sig clusters show some degree of expression of homeostatic and DAM gene sets. Our cluster hub classifications are relative based on changes in gene expression levels and changes in the percentage of cells that express related marker genes. For example, while the homeostatic-enriched cluster hub is defined based on a relatively enriched expression of homeostatic genes and a relatively low expression of some DAM genes, given the high expression of other core DAM genes, this does not necessarily mean that cells in the MG-sig homeostatic-enriched cluster hub are transcriptionally comparable to homeostatic microglia in the healthy brain of a young adult mouse.

Cluster 14, which transcriptionally aligned with both microglial and monocyte/macrophage profiles, highly expressed several genes associated with DAM-B microglia (e.g., *Trem2*, *Cd68*, *Cst7*, *Clec7a*, and *Fabp5*). While Mye-sig clusters also expressed high levels of DAM genes, including *Fth1*, *Tyrobp*, and *Apoe*, the Mye-sig clusters displayed large increases in the monocyte/macrophage and DAM marker gene *Lgals3* and showed little expression of the remaining DAM genes, including *Trem2* (Figure 4).

### 3.4. SCF+G-CSF Treatment Increases the Proportion of Mye-sig Cells in the Brains of Aged APP/PS1 Mice

**Figure 5:**
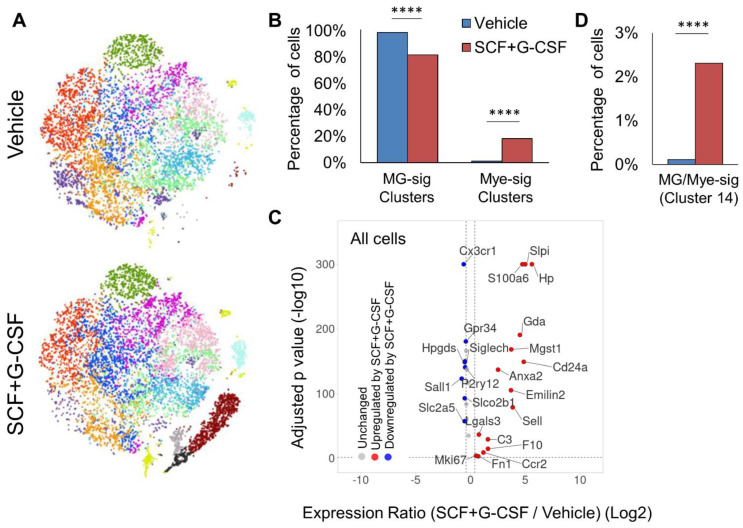
SCF+G-CSF treatment alters the distribution of cells across clusters, augmenting the prevalence of Mye-sig cells in the brains of aged APP/PS1 mice. (**A**) t-distributed stochastic neighbor embedding (tSNE) dimensionality reduction plots of cells separated by treatment. (**B**) SCF+G-CSF treatment shifts the distribution of CD11b^+^ cells with reduced proportions of MG-sig cells and increased proportions of Mye-sig cells in the brains of APP/PS1 mice. **** *p* < 0.0001. (**C**) A volcano plot highlights differential expression across SCF+G-CSF-treated and vehicle-treated groups of MG-sig and Mye-sig cell gene sets. Genes upregulated by SCF+G-CSF treatment are shown in red. Genes downregulated by SCF+G-CSF treatment are shown in blue. False discovery rate-corrected *p* values (−log10) are shown on the y-axis. The log2-transformed expression ratios are computed as expression levels in the SCF+G-CSF-treated mice compared to those in the vehicle control group and are plotted on the x-axis. Dotted lines indicate significance thresholds. (**D**) SCF+G-CSF treatment increases the number of MG/Mye-sig (cluster 14) cells. **** *p* < 0.0001.

Next, we sought to determine whether SCF+G-CSF treatment alters the proportions of MG-sig and Mye-sig cells in the brains of aged APP/PS1 mice. Figure 5A shows t-SNE plots for visualizing cell clusters in the vehicle control and SCF+G-CSF treatments. In the vehicle controls, 98.8% of the cells were contained in the MG-sig clusters (clusters 1–8 and 10), and 1.2% in the Mye-sig clusters (clusters 9, 12, and 13) (Figure 5B and Figure S3). Cell distributions in the SCF+G-CSF-treated mice were shifted, with a significantly reduced percentage of cells in the MG-sig clusters (81.8% of cells; *p* < 0.0001) and a significantly increased percentage of cells in the Mye-sig clusters (18.2% of cells; *p* < 0.0001) as compared to those in the vehicle controls (Figure 5B and Figure S3). Strikingly, cells from the SCF+G-CSF-treated mice accounted for 95.1% (range across clusters: 82–99%) of the cells contained in the Mye-sig clusters (9, 12, and 13; Figure S3B). In line with these findings, the expression levels of all monocyte/macrophage gene markers were significantly upregulated by SCF+G-CSF treatment (Figure 5C). We also observed that the cluster 14 cells, which highly co-expressed microglial and monocyte/macrophage gene profiles, were almost exclusively (96.4%) from the SCF+G-CSF-treated mice (Figure 5D and Figure S3B). Altogether, these findings suggest that SCF+G-CSF treatment may enhance the recruitment of myeloid cells into the brains of aged APP/PS1 mice.

**Figure 6:**
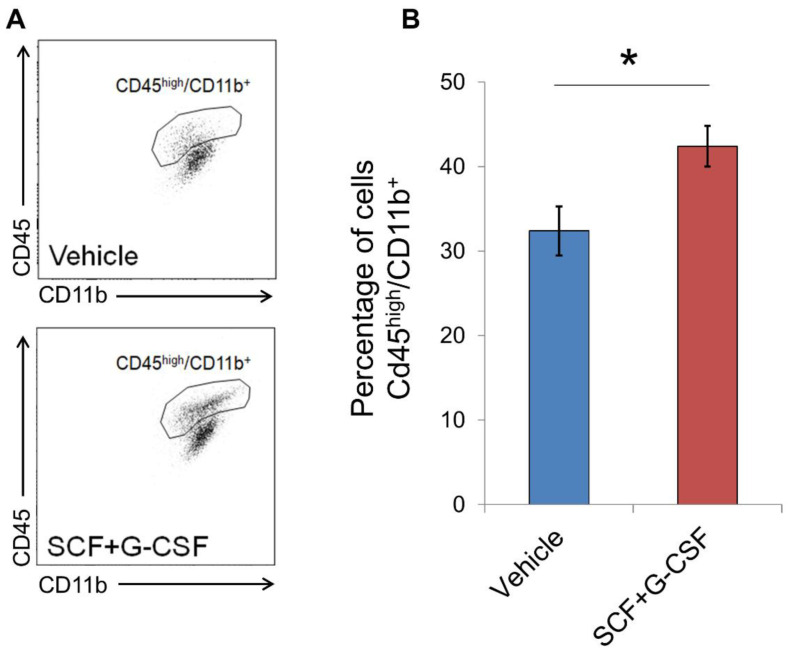
SCF+G-CSF treatment augments the recruitment of CD11b^+^/CD45^high^ cells into the brains of aged APP/PS1 mice. (**A**) Representative flow cytometry scatter plots show the population of CD45^high^/CD11b^+^ cells, thought to largely reflect peripherally derived myeloid cells/active phagocytes, in the brains of aged APP/PS1 mice treated with or without SCF+G-CSF. (**B**) SCF+G-CSF treatment increases the percentage of cells showing a CD45^high^/CD11b^+^ profile in the brains of aged APP/PS1 mice. Data are presented as mean ± SEM. * *p* < 0.05; *t*-test, n = 4 in each group.

To validate the scRNAseq data that suggest that SCF+G-CSF treatment increases the population of peripheral myeloid cells in the brain, we measured the cell surface expression of CD11b and CD45 using flow cytometry in separate aliquots of MACS-isolated CD11b^+^ cells. Previous studies show that the surface expression profiles of these proteins can be used to differentiate myeloid cells (CD11b^+^/CD45^high^) from microglia in the brain [90,91]. We found that SCF+G-CSF treatment significantly increased the proportion of CD11b^+^/CD45^high^ cells in the brains of aged APP/PS1 mice compared to the vehicle controls (*p* < 0.05, Figure 6). These data support the scRNAseq findings suggesting that SCF+G-CSF treatment increases the population of peripherally derived myeloid cells or CD11b^+^/CD45^high^ active phagocytes in the brains of aged APP/PS1 mice.

### 3.5. SCF+G-CSF Treatment Changes CD11b^+^ Cell Activation States in the Brains of Aged APP/PS1 Mice

We next assessed the effects of SCF+G-CSF treatment in modifying the activation profiles in CD11b^+^ MG-sig clusters. SCF+G-CSF treatment significantly increased the percentage of MG-sig cells contained in the DAM-A hub (*p* < 0.0001, Figure S4) and reduced the percentage of MG-sig cells in the hybrid cluster (i.e., cluster 10, which showed a relatively high expression of DAM, inflammatory, and homeostatic marker genes) (*p* < 0.0001, Figure S4). The proportions of MG-sig cells contained in the homeostatic-enriched hub and DAM-B hub were not changed by SCF+G-CSF treatment (Figure S4). These results suggest a shift away from inflammatory and homeostatic profiles in DAM-associated cells following SCF+G-CSF treatment. Consistent with these findings, differential expression analysis across all MG-sig clusters revealed that SCF+G-CSF treatment reduced the expression of inflammatory markers (e.g., *Nfkbiz*, *Tnf*, and *Il1b*; Figure S5A). However, SCF+G-CSF treatment also reduced the expression of gene sets typically upregulated in DAM (e.g., *Csf1*, *Axl*, and *Itgax*) as well as those typically downregulated in DAM (e.g., *Egr1*, *Sall1*, and *Jun*; Figure S5A), suggesting a variable or nuanced modification of the transcriptional response by SCF+G-CSF treatment in DAM gene sets. These results were largely consistent upon comparison of gene sets across all clusters (Figure S5B), with the notable exception of *Il1b*. *Il1b* was upregulated with SCF+G-CSF treatment (when pooling all clusters), an effect primarily driven by *Il1b* increases in the treatment-associated Mye-sig clusters (see Figure 4).

### 3.6. The Most Prominent Transcriptome-Wide Responses to SCF+G-CSF Treatment Are Largely Comparable in MG-sig and Mye-sig Clusters in the Brains of Aged APP/PS1 Mice

**Figure 7:**
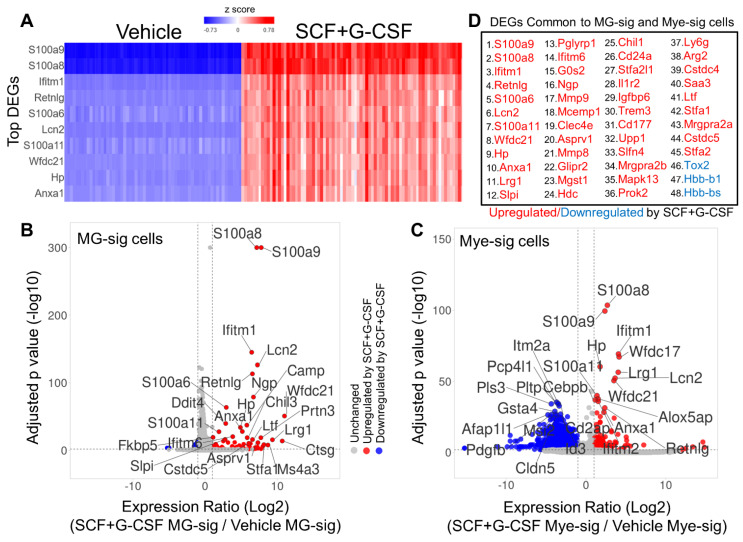
Transcriptome-wide responses to SCF+G-CSF treatment in MG-sig and Mye-sig cells in the brains of aged APP/PS1 mice. (**A**) A hierarchical clustering heat map illustrates the top 10 differentially expressed genes that are upregulated (in red) by SCF+G-CSF treatment across all cell clusters pooled together. The two upregulated genes *S100a8* and *S100a9* show the strongest effect following SCF+G-CSF treatment. (**B**,**C**) Volcano plots highlight genes differentially expressed by SCF+G-CSF treatment in MG-sig clusters (**B**) and in Mye-sig clusters (**C**). Genes upregulated by SCF+G-CSF treatment are shown in red. Genes downregulated by SCF+G-CSF treatment are shown in blue. In MG-sig cells, 70 genes are upregulated and 18 genes are downregulated by SCF+G-CSF. In Mye-sig cells, 133 genes are upregulated and 2637 genes are downregulated by SCF+G-CSF. False discovery rate-corrected *p* values (−log10) are shown on the y-axis. The log2-transformed expression ratios are computed as expression levels in SCF+G-CSF-treated mice relative to those in the vehicle control group. The log2-transformed expression ratios are plotted on the x-axis. Dotted lines indicate inclusion thresholds: ≥2-fold change, and FDR-corrected *p* value < 0.01. Note, *p* values are restricted to 300 decimal places. The top 25 genes differentially expressed are labeled. (**D**) A list of genes significantly altered (≥2-fold change, FDR-corrected *p* value < 0.01) by SCF+G-CSF treatment that are common to both MG-sig and Mye-sig cells is presented. Genes are ordered from lowest to highest FDR-corrected *p* value in the entire dataset.

Next, we performed transcriptome-wide differential gene analysis across experimental groups for unbiased identification of transcriptional changes in MG-sig clusters and Mye-sig clusters following SCF+G-CSF treatment. We first identified genes altered by SCF+G-CSF treatment when pooling all cell clusters together. We observed that the vast majority of differentially expressed genes (DEGs) was upregulated (n = 359 genes) by SCF+G-CSF treatment, rather than downregulated (n = 34 genes; Figure S6). The top 10 DEGs ranked by significance and the number of differentially expressed transcripts (Figure 7A) included four genes of the calcium-binding S100a family (*S100a8*, *S100a9*, *S100a6*, and *S100a11*), the lipocalin family gene *Lcn2*, the anti-inflammatory annexin A1 (*Anxa1*), the hemoglobin-binding haptoglobin (*Hp*), the anti-viral interferon-induced transmembrane gene *Ifitm1*, the resistin-like molecule *Retnlg*, and the whey acidic protein/four-disulfide core domain 21 (*Wfdc21*). These 10 genes were all upregulated by SCF+G-CSF treatment. This gene set collectively plays a prominent role in immune cell activation processes, such as microglia- and macrophage-mediated inflammation, phagocytosis, and cell migration. Gene set enrichment analysis corroborated these findings linking the DEGs with immune responses, inflammatory responses, stress responses, and leukocyte migration (Figure S7; Supplemental Data File S2).

The topmost genes upregulated by SCF+G-CSF treatment were largely comparable across MG-sig (Figure 7B) and Mye-sig (Figure 7C) clusters and across individual MG-sig cluster hubs (Figure S8). These findings identify a stable transcriptional response following SCF+G-CSF treatment in brain immune cells of aged APP/PS1 mice. The expression profiles across individual cell clusters for the top 25 genes upregulated by SCF+G-CSF treatment are presented in Figure S9. The overlapping genes (n = 48) prominently altered by SCF+G-CSF treatment in both MG-sig and Mye-sig clusters (Supplemental Data File S3) are presented in Figure 7D.

While many of the DEGs upregulated by SCF+G-CSF treatment showed consistent profiles in MG-sig and Mye-sig clusters, we also noted distinct SCF+G-CSF-related transcriptional profiles across clusters. The majority of DEGs prominently altered by SCF+G-CSF treatment in MG-sig clusters were upregulated (n = 70 genes) rather than downregulated (n = 18 genes). By contrast, in Mye-sig clusters, 133 genes were upregulated and 2637 genes were downregulated (Figure 7B,C). These results suggest a more extensive transcriptional response to SCF+G-CSF treatment in the Mye-sig cells. Of note, the topmost genes downregulated by SCF+G-CSF in Mye-sig clusters included those associated with actin remodeling, cell adhesion and migration (e.g., *Cd2ap*, *Pls3*, *Myo10*, and *Afap1l1*), vascular integrity (e.g., *Pdgfb* and *Cldn5*), and lymphocyte activation (e.g., *Itm2a* and *Cd2ap*).

### 3.7. S100a8 and S100a9 Are Robustly Expressed in MG-sig Clusters and Mye-sig Clusters in Aged APP/PS1 Mice following SCF+G-CSF Treatment

**Figure 8:**
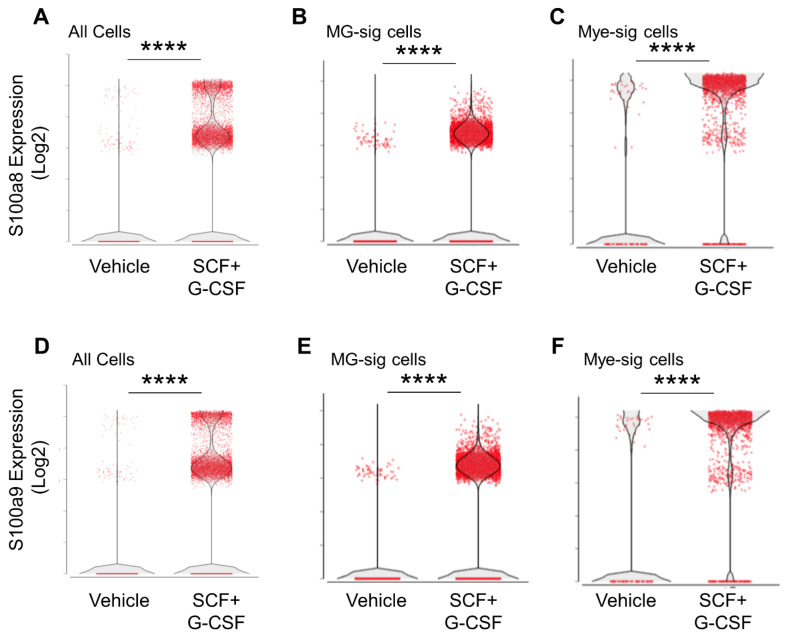
S100a8 and S100a9 are robustly upregulated by SCF+G+CSF treatment in aged APP/PS1 mice and most highly expressed in Mye-sig clusters. Violin density plots show that S100a8 (**A**–**C**) and S100a9 (**D**–**F**) are significantly upregulated by SCF+G-CSF treatment in cells pooled together across all clusters (**A**,**D**), in MG-sig clusters (**B**,**E**), and in Mye-sig clusters (**C**,**F**). Individual cells are shown as red dots. **** *p* < 0.0001.

Overall, the most striking effect following SCG+G-CSF treatment was the upregulation of the genes *S100a8* and *S100a9*. As *S100a8* and *S100a9* correlate with and interact directly with Aβ plaques, and they both are highly expressed in microglia and macrophages that surround plaques [92,93], we further probed the *S100a8* and *S100a9* expression profiles in our dataset. Violin density plots show the distributions of *S100a8* and *S100a9* expression in MG-sig and Mye-sig cells in the vehicle control and SCF+G-CSF treatment groups (Figure 8). The vast majority of the CD11b^+^ cells that highly expressed *S100a8* or *S100a9* were observed in the SCF+G-CSF-treated mice, with the highest expression in the Mye-sig cells of the SCF+G-CSF-treated mice.

To ensure that SCF+G-CSF treatment upregulated *S100a8* and *S100a9* (*S100a8/9*) expression in MG-sig cells rather than in small subsets of Mye-sig cells contained in the MG-sig clusters, we probed the expression levels of microglial gene markers in *S100a8/9*-positive cells and *S100a8/9*-negative cells. In MG-sig clusters, *S100a8/9*-positive cells co-expressed several specific gene markers of microglia, including Tmem119, at comparable levels to those of *S100a8/9*-negative cells (Figure S10). This observation suggests that while the expression of *S100a8/9* is heightened in Mye-sig cells (see Figure 8), the genes are also robustly enhanced in MG-sig cells following SCF+G-CSF treatment.

Interestingly, the topmost genes upregulated with SCF+G-CSF treatment in the entire dataset were co-expressed and upregulated in the *S100a8/9*-positive MG-sig cells compared to the *S100a8/9*-negative MG-sig cells, as well as in the *S100a8/9*-positive Mye-sig cells compared to the *S100a8/9*-negative Mye-sig cells (Figure S11). These findings suggest that *S100a8* and *S100a9* are reliable marker genes associated with the prominent transcriptional responses to SCF+G-CSF treatment in brain immune cells in aged APP/PS1 mice.

### 3.8. Network Analysis of DEGs across Experimental Groups Identifies Highly Inter-Connected “hub” Genes Functionally Connected to S100a8/9 Expression

**Figure 9:**
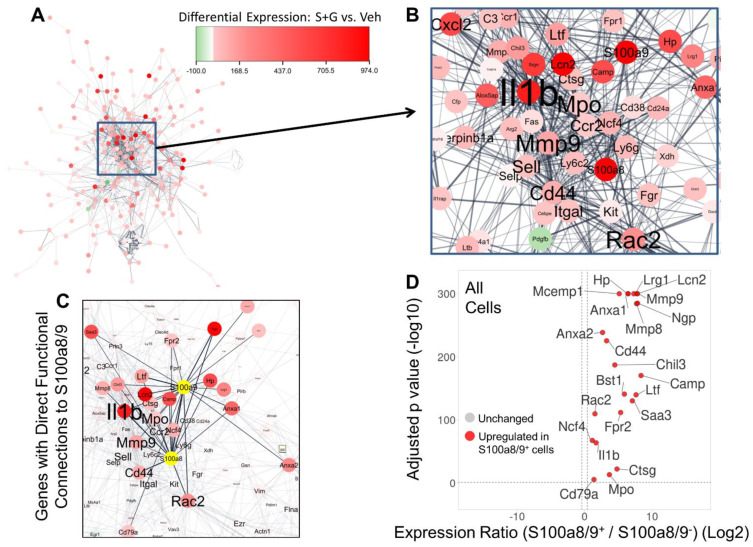
Functional network of genes differentially expressed by SCF+G-CSF treatment in aged APP/PS1 mice reveals highly inter-connected “hub” genes and those functionally linked with *S100a8* and *S100a9*. (**A**) The genes significantly altered by SCF+G-CSF treatment (when all cells are pooled together) were used to create a functional gene-gene interaction network by querying their gene products in the STRING database. Each node corresponds to a gene significantly altered by SCF+G-CSF treatment. The edges (or links) between the nodes correspond to confirmed or potential functional connections. The color of the node corresponds to the intensity of differential expression, with red color indicating upregulation by SCF+G-CSF treatment. (**B**) A central hub of genes includes several of the top upregulated genes with SCF+G-CSF treatment (e.g., *S100a8*, *S100a9*, *Anxa1*, *Lcn2*, and *Hp*). The size of the gene font corresponds to the extent of its connectivity in the network, identifying highly inter-connected “hub” genes, including *Il1b*, *Mmp9*, *Rac2*, and *Cd44*. (**C**) The 22 genes (*Anxa1*, *Anxa2*, *Bst1*, *Camp*, *Cd44*, *Cd79a*, *Chil3*, *Ctsg*, *Fpr2*, *Hp*, *Il1b*, *Lcn2*, *Lrg1*, *Ltf*, *Mcemp1*, *Mmp8*, *Mmp9*, *Mpo*, *Ncf4*, *Ngp*, *Rac2*, and *Saa3*) directly connected to *S100a8* and *S100a9* (*S100a8/9*) are highlighted. (**D**) This *S100a8/9*-linked gene set identified by network analysis is largely co-expressed and dramatically upregulated in individual *S100a8/9*-positive vs. *S100a8/9*-negative cells in the single-cell RNA sequencing dataset. Genes upregulated in *S100a8/9*-positive cells are shown in red. False discovery rate-corrected *p* values (−log10) are shown on the y-axis. The log2-transformed expression ratios are computed as expression levels in *S100a8/9*-positive cells relative to those in *S100a8/9*-negative cells and are plotted on the x-axis. Dotted lines indicate significance thresholds. Note, *p* values are restricted to 300 decimal places.

To identify potential interactions among the genes altered by SCF+G-CSF treatment, we constructed a functional network of gene–gene interactions among treatment-related DEGs (Figure 9). A central hub within the network contained several of the top DEGs, including *S100a8/9*. Within this hub, we also noted highly inter-connected genes, including *Il1b*, *Mmp9*, *Mpo*, *Cd44*, and *Rac2* (Figure 9A,B). These findings suggest that the central functions of CD11b^+^ cells changed by SCF+G-CSF treatment are associated with inflammation-like activation (*Il1b*, *Mpo*, and *Cd44*) and cell motility and remodeling (*Mmp9*, *Rac2*, and *Cd44*).

Twenty-two genes (*Anxa1*, *Anxa2*, *Bst1*, *Camp*, *Cd44*, *Cd79a*, *Chil3*, *Ctsg*, *Fpr2*, *Hp*, *Il1b*, *Lcn2*, *Lrg1*, *Ltf*, *Mcemp1*, *Mmp8*, *Mmp9*, *Mpo*, *Ncf4*, *Ngp*, *Rac2*, and *Saa3*) upregulated by SCF+G-CSF treatment were directly connected to *S100a8/9* (Figure 9C), suggesting direct functional interactions. Importantly, *S100a8/9*-positive MG-sig and Mye-sig cells showed an enriched expression of all 22 of these *S100a8/9*-connected genes (Figure 9D; Figure S12). We note that the expression of the hub gene *Il1b* was unchanged in *S100a8/9*-positive MG-sig cells (Figure S12A). However, the expression of *Il1b* was upregulated by SCF+G-CSF treatment when all cells were pooled together (Figure S5B) and in *S100a8/9*-positive Mye-sig cells (Figure S12B). These findings demonstrate select contributions of *S100a8/9*-positive Mye-sig cells to SCF+G-CSF-related increases in the inflammatory hub gene *Il1b*.

Gene set enrichment analysis of *S100a8/9*-connected genes identified by network analysis showed enriched functions related to immune responses, inflammation (*Cd44*, *Saa3*, *Anxa1*, *Il1b*, *Fpr2*, *Hp*, *Camp*, *S100a8*, *S100a9*, *Bst1*, and *Mmp8*), and leukocyte migration (*Mmp9*, *Anxa1*, *Il1b*, *Rac2*, *Fpr2*, *Bst1*, *S100a8*, and *S100a9*) (Figure S13). These findings are consistent with gene set enrichment analyses run on all DEGs (Figure S7). Additionally, enriched Reactome pathways included degradation of the extracellular matrix (*Mmp9*, *Mmp8*, *Ctsg*, and *Cd44*), implicating a mechanism related to cell motility and migration associated with SCF+G-CSF treatment. A complete list of enriched biological processes and Reactome pathways is provided in Supplemental Data File S2.

## 4. Discussion

In the present study, we performed a transcriptome-wide analysis of CD11b^+^ microglia and myeloid cells in the brains of 28-month-old APP/PS1 mice at the single-cell level following systemic treatment with SCF+G-CSF. Most notably, the findings of this study show that SCF+G-CSF treatment (1) increases the proportions of brain immune cells that align transcriptionally with peripherally derived monocytes/macrophages, and (2) induces transcriptional responses in brain immune cells that are associated with cell activation, cell migration, inflammation, and Aβ clearance. These findings provide insight into how SCF+G-SCF treatment may modulate microglia and peripherally derived myeloid cells to reverse or mitigate neuropathology in the context of AD in old age.

The majority of the cell clusters transcriptionally aligned with microglia of distinct activation states. These findings are in line with prior reports showing that the vast majority of CD11b^+^ brain cells are microglia comprising transcriptionally heterogeneous sub-populations in the aged, injured, or AD brain [94,95,96,97,98]. While all MG-sig clusters expressed high levels of signature DAM genes, between-cluster variability in MG-sig cells was largely characterized by an inverse relationship between homeostatic and DAM profiles. This finding is well aligned with those of previous studies showing a transcriptional heterogeneity of microglia in AD that reflects transitionary stages from a relatively homeostatic to a highly reactive disease-associated phenotype [72,98].

SCF+G-CSF treatment increased the proportion of DAM-like cells marked by relatively little expression of certain homeostatic genes (e.g., P2ry12). A microglial phenotype characterized by reductions of P2ry12 has been found next to Aβ plaques in the brains of AD patients [99]. Moreover, our earlier study showed that P2ry12 expression is reduced in subsets of microglia that closely associate with Aβ plaques in the brains of aged APP/PS1 mice following SCF+G-CSF treatment [22]. Therefore, our findings are consistent with an effect of SCF+G-CSF to augment DAM-like cells in the brains of aged APP/PS1 mice with an excessive amount of Aβ plaques. Additionally, we found that SCF+G-CSF treatment reduced pro-inflammatory gene expression (e.g., *Il1b* and *Tnf*) in MG-sig clusters. Reductions in inflammatory genes in DAM are associated with enhanced phagocytosis of Aβ [89]. Taken together, the SCF+G-CSF-increased DAM transcriptional profiles reported here may reflect increases in subsets of highly phagocytic but less inflammatory microglia that may lead to increases of Aβ clearance. These effects may contribute to a SCF+G-CSF-reduced Aβ burden and reduced neuroinflammation in aged APP/PS1 mice [22]. Similar effects are observed following granulocyte macrophage colony-stimulating factor (GM-CSF) treatment. GM-CSF augments the number of reactive Iba1^+^ cells localized to Aβ plaques, reduces inflammation, and enhances clearance of Aβ deposition [100,101].

SCF+G-CSF treatment increased the proportion of myeloid cells in the brains of aged APP/PS1 mice. The vast majority of cell clusters with Mye-sig transcriptional profiles were found in the SCF+G-CSF-treated mice, a finding consistent with SCF+G-CSF-increased CD11b^+^/CD45^high^ peripheral myeloid cells or active phagocytes [102] in the brains of aged APP/PS1 mice. While our transcriptomic and flow cytometry experiments suggest the same directionality of effect (i.e., an increase in peripherally derived myeloid cells following SCF+G-CSF treatment), the percentage of cells with a CD11b^+^/CD45^high^ profile in the flow cytometry experiment was higher than the percentage of cells in the Mye-sig clusters identified by transcriptomic profiling. It is possible that distinct pre-processing methods contribute to these differences. Additionally, it is possible that a subset of activated microglia that upregulate CD45 [81,102] could best align with a microglial transcriptional signature in the scRNAseq experiment and have a CD45^high^ immunophenotype in the flow cytometry experiment.

Accumulating evidence suggests a prominent role of infiltrating myeloid cells in mitigating Aβ pathology in AD [31,32,33,103,104]. GM-CSF treatment has been shown to increase monocyte migration [105], reduce Aβ plaques, and reverse cognitive deficits [100,101,106]. Likewise, our previous findings revealed that SCF+G-CSF treatment increases the association of bone marrow-derived macrophages with Aβ plaques and reduces Aβ deposition in APP/PS1 mice [71]. In addition, our unpublished two-photon live brain imaging study reveals an acute effect of SCF+G-CSF in reducing Aβ plaques during a 7-day treatment in APP/PS1 mice. Together with the observation of the current study showing SCF+G-CSF-increased CD11b^+^/CD45^high^ myeloid cells or active phagocytes [102] in the brains of aged APP/PS1 mice following a 5-day treatment of SCF+G-CSF, these findings point to a crucial role for peripherally derived myeloid cells in SCF+G-CSF-enhanced Aβ clearance in the aged brains of APP/PS1 mice.

The top genes upregulated by SCF+G-CSF treatment (e.g., *S100a8*, *S100a9*, *Ifitm1*, *Lcn2*, and *Mmp9*) are associated with activated microglia and infiltrating myeloid cells, inflammation, and cell migration to sites of injury [92,107,108,109,110,111,112]. Network analysis confirmed these effects by identifying hub genes that are functionally related to cell movement and remodeling (*Mmp9*, *Rac2,* and *Cd44*) and inflammation (*Il1b*, *Mpo*, and *Cd44*).

*S100a8* and *S100a9* were robustly upregulated by SCF+G-CSF. *S100a8/9*-positive microglia and peripheral myeloid cells highly co-expressed the topmost differentially upregulated genes after SCF+G-CSF treatment, suggesting a conserved or core transcriptional profile related to SCF+G-CSF treatment. The S100a family genes encode calcium-binding proteins that regulate fundamental processes, including intracellular calcium homeostasis and cytoskeleton rearrangement. The secretion of S100a8 and S100a9 is associated with inflammatory processes [107,113,114], and S100a9 is essential for the trans-endothelial migration of leukocytes [107,115]. Importantly, S100a8/9 are enriched in the brains and sera of AD patients and are thought to contribute to AD pathogenesis [92,107,116]. Given these findings, we were surprised to find that *S100a8/9* were the topmost upregulated genes following SCF+G-CSF treatment.

Relevant to our findings, inflammation and Aβ plaque load enhance *S100a8/9* transcription and secretion by microglia and macrophages [92,93]. Therefore, findings that SCF+G-CSF treatment increases the numbers of activated microglia and macrophages that engulf Aβ plaques [22,71] is consistent with SCF+G-CSF-enhanced *S100a8/9* transcription. Interestingly, it has been shown that S100a9, while accelerating plaque formation, may also reduce neural toxicity and inflammation caused by Aβ [117,118]. While secreted S100a8/9 can induce inflammation [107], high levels of extracellular calcium induce the formation of S100a8/9 heterotetramers, which may reduce inflammatory tone [119]. These findings are in line with the anti-inflammatory properties of S100a8/9 under certain conditions [107]. In the current study, SCF+G-CSF treatment induced the downregulation of inflammatory *Il1b* and *Tnf* and the robust upregulation of *S100a8/9* in MG-sig cells, suggesting a potential role for *S100a8/9* in modulating inflammatory signaling in aged APP/PS1 mice.

While it remains possible that enhancement of inflammatory cytokines may contribute to phagocytic clearance of Aβ [23,47,48,49,50] following SCF+G-CSF treatment, the inflammation associated with S100a8/9 may also be mitigated by anti-inflammatory-mediating genes co-upregulated by SCF+G-CSF treatment in microglia and peripherally derived myeloid cells. Several genes that are highly co-expressed in S100a8/9-positive MG-sig and Mye-sig cells or functionally linked with S100a8/9 expression (e.g., *Anxa1*, *Anxa2*, *Chil3*, *Hp*, *Lrg1*, *Ngp*, and *Wfdc17*) confer anti-inflammatory properties [120,121,122,123,124,125,126,127,128] associated with tissue repair [39,43,129]. Additionally, several of the genes (e.g., *Anxa1*, *Anxa2*, *Fpr2*, *S100a6*, *S100a11*, *Mmp9*, *Hp*, and *Il1b*) robustly upregulated by SCF+G-CSF treatment and linked with *S100a8/9* are also associated with Aβ clearance and/or neuroprotection. These genes and their roles in AD and neuroprotection are briefly outlined below.

Microglial Anxa1 mediates the non-inflammatory phagocytosis of apoptotic cells, and under inflammatory conditions, Anxa1 restores the non-inflammatory clearance of apoptotic neurons [128]. Anxa1 treatment in vitro enhances Aβ phagocytosis by microglia and reduces the Aβ-stimulated transcription of inflammatory genes [130]. In young 5xFAD mice, Anxa1 reduces Tnf, mitigates vascular pathology, and increases synaptic densities [131]. The effects of Anxa1 in Aβ clearance are largely mediated by activation of its Fpr1/2 receptor [130], while a neuroprotective role for Anxa1-S100a11 interactions is also reported [132]. SCF+G-CSF treatment upregulated the expression of *Anxa1*, *Fpr2*, and *S100a11*. It has been also shown that Anxa2 plays a role in anti-inflammation and neuroprotection [126].

S100a6 binds calcium and zinc and is found in cells that surround Aβ plaques. In a study targeting the contribution of zinc to Aβ aggregation, S100a6 treatment reduced Aβ plaques in the brains of aged APP/PS1 mice [133].

*Hp* encodes haptoglobin, a hemoglobin-binding protein. Its scavenging effects prevent neurotoxicity in sub-arachnoid hemorrhage [134]. As cerebral micro-bleeds are associated with AD and Aβ burden [135], SCF+G-CSF-increased Hp expression may mitigate AD-induced AD neuropathology compounded by vascular pathology. Haptoglobin may also directly inhibit the formation of Aβ fibrils [136], and sequesters high-mobility group box-1 (HMGB1) [127], which activates neuroinflammation and inhibits Aβ clearance [137,138].

The endopeptidase Mmp9 plays key roles in extracellular matrix remodeling, cell migration, and neuroplasticity [139]. Mmp9 is increased in AD and can degrade Aβ plaques [140,141]. Neuronal over-expression of Mmp9 in 5xFAD mice shifts APP processing toward an α-secretase pathway, coinciding with enhanced presynaptic densities [142]. An α-secretase pathway generates soluble APP-α, a non-amyloidogenic peptide [143,144].

The pro-inflammatory cytokine Il1b is associated with Aβ clearance, and Il1b increases Iba1^+^ microglia and macrophage densities [49,50]. Our findings show that SCF+G-CSF treatment increased Il1b expression in Mye-sig clusters, while reducing Il1b in MG-sig clusters. It remains unclear if Mye-sig-derived Il1b may enhance Aβ plaque removal.

Many of the genes upregulated by SCF+G-CSF treatment (e.g., *S100a8*, *S100a9*, *S100a11*, *S100a6*, *Lcn2*, *Hp*, *Mmp8*, *Mmp9*, *Anxa1*, *Anxa2*, *Fth1*, and *Ltf*) are activated by or modulate the concentrations of calcium, zinc, and/or iron. Metal sequestration and response to metal ions were enriched pathways associated with SCF+G-CSF. As metal ion concentrations are linked to AD pathogenesis [145,146], our findings support further research into metal sequestration functions following SCF+G-CSF treatment in the context of AD.

While future studies are needed to determine whether the treatment-related gene sets outlined here play causal roles in resolving AD pathology, we note that the robust transcriptional changes induced by SCF+G-CSF treatment remarkably overlap with those in brain CD11b^+^ cells during a period of functional recovery in a mouse model of TDP-43 proteinopathy [147]. It remains to be determined whether this upregulated gene set could serve as a transcriptional marker of brain repair in distinct pathologies.

There are several limitations of our study. Microglia-like (MG-sig) and myeloid-like (Mye-sig) cell populations were identified based on gene sets shown to differentiate the two cell classes [79]. If severe neuropathology in the aged APP/PS1 mouse brain, and/or the SCF+G-CSF treatment, significantly altered the expression profiles of our marker gene sets, our cell type classifications may be limited. It is possible that our cell clusters represent mixed populations of microglial and myeloid cells. Moreover, several myeloid cell classes can infiltrate the brains of transgenic mouse models of AD [148,149]. Monocytes/macrophages are the primary bone marrow-derived cell population in the AD brain [31,150], showing a dramatic influx following SCF+G-CSF treatment [71]. However, further work is needed to classify the populations of infiltrating myeloid cells following SCF+G-CSF treatment in the aged APP/PS1 brain. Also, the use of tissue from a young, healthy, control mouse would better facilitate the classification of activation states (e.g., disease-associated and homeostatic). Corroboration of the transcriptional responses to SCF+G-CSF in identified subsets of cell classes using immunohistochemistry and/or single-molecule in situ hybridization will prove useful to validate our findings.

We evaluated brain-wide transcriptional profiles of CD11b^+^ cells. As AD neuropathologies are spatially diverse [151], it is possible that our study did not uncover effects limited to select brain regions. We further stress that this scRNAseq study was run on pooled samples of APP/PS1 mice. Therefore, we were unable to distinguish the contributions of individual samples to the related transcriptional profiles.

In conclusion, the findings of this scRNAseq study reveal potential cellular and molecular mechanisms by which systemic treatment of SCF+G-CSF modulates microglia and peripherally derived myeloid cells to mitigate AD pathology in the aged APP/PS1 mouse brain. Future studies will clarify the causal roles of the identified candidate mechanisms.

## Data Availability

The original contributions presented in the study are included in the article/Supplementary Material, further inquiries can be directed to the corresponding author.

## Supplementary Materials

The following supporting information can be downloaded at: https://www.mdpi.com/article/10.3390/biom14070827/s1, Figure S1. Cell clusters highly express marker genes of microglia and macrophages but not marker genes of off-target cells in the brain. Figure S2. Myeloid cell marker genes and microglial marker genes are respectively enriched in Mye-sig and MG-sig cell clusters. Figure S3. The percentage of cells contained in individual clusters across experimental groups. Figure S4. SCF+G-CSF treatment alters the reactive profiles of MG-sig cells. Figure S5. SCF+G-CSF treatment modestly alters gene sets associated with homeostatic microglia, DAM, and inflammatory microglia. Figure S6. Transcriptome-wide responses to SCF+G-CSF treatment. Figure S7. Gene ontology analysis of all DEGs upregulated by SCF+G-CSF treatment. Figure S8. Transcriptome-wide responses to SCF+G-CSF treatment in MG-sig cluster hubs. Figure S9. Cluster-specific expression of the topmost differentially expressed genes upregulated by SCF+G-CSF treatment. Figure S10. Microglial cell-selective genes are comparably expressed in S100a8/9-positive and S100a8/9-negative MG-sig cells. Figure S11. S100a8/9-positive MG-sig and Mye-sig cells highly and differentially express the topmost differentially expressed genes upregulated by SCF+G-CSF treatment. Figure S12. Genes identified by network analysis to have direct functional connections to S100a8/9 are largely upregulated in S100a8/9-positive MG-sig cells and S100a8/9-positive Mye-sig cells. Figure S13. Gene ontology analysis of genes with direct functional connections to S100a8/9. File S1. Mean expression (log2) of all genes organized by cluster. File S2. A complete list of enriched terms from the GO Biological Process and Reactome databases is provided from separate analyses on all genes and on those functionally connected to S100a8/9 as identified by network analysis. File S3. A list is provided of the genes differentially expressed in SCF+G-CSF treatment vs. vehicle controls that are common to both MG-sig and Mye-sig clusters. Table S1. Individual cells in cluster 14 co-express marker genes for microglia and myeloid cells. Table S2. Individual cells in cluster 10 highly co-express marker genes of homeostatic microglia and reactive or disease-associated microglia (DAM).

## List of Abbreviations

Aβ	Amyloid-beta
AD	Alzheimer’s disease
DAM	Disease associated microglia
DEGs	Differentially expressed genes
D-PBS	Dulbecco’s phosphate-buffered saline
FDR	False discovery rate
G-CSF	Granulocyte colony-stimulating factor
MACS	Magnetic-activated cell sorting
MG-sig	Microglial transcriptional signature
Mye-sig	Peripherally derived myeloid transcriptional signature
scRNAseq	Single-cell RNA sequencing
SCF	Stem cell factor
TBI	Traumatic brain injury
tSNE	t-distributed stochastic neighbor embedding
UMIs	Unique molecular identifiers
