# Supplementary material for: Single-Cell RNA Sequencing Reveals Immunomodulatory Effects of Stem Cell Factor and Granulocyte Colony-Stimulating Factor Treatment in the Brains of Aged APP/PS1 Mice
Source: Biomolecules. 2024 Jul 10;14(7):827. doi: 10.3390/biom14070827 (PMC11275138; doi:10.3390/biom14070827)
Supplement: Supplementary file 1 [file biomolecules-14-00827-s001.zip › Supplemental data/Files/Supplemental Data File Legends.pdf]

Supplemental Data Files

Supplemental Data File S1. Mean expression (log2) of all genes organized by cluster. Rows correspond to individual genes. Columns correspond to individual clusters.

Supplemental Data File S2. A complete list of enriched terms from the GO Biological Process and Reactome databases is provided from separate analyses on all genes and on those functionally connected to S100a8/9 as identified by network analysis. Results using the two gene sets are presented in separate tabs.

Supplemental Data File S3. A list is provided of the genes differentially expressed in SCF+G-CSF treatment vs. vehicle controls that are common to both MG-sig and Mye-sig clusters. Genes included in the list show ≥ 2-fold change in SCF+G-CSF treatment compared to vehicle controls with an FDR-corrected *p*-value < 0.01. Differential expression values and adjusted *p* values are provided.
