# Supplementary figures and images for: Single-Cell RNA Sequencing Reveals Immunomodulatory Effects of Stem Cell Factor and Granulocyte Colony-Stimulating Factor Treatment in the Brains of Aged APP/PS1 Mice
Source: Biomolecules. 2024 Jul 10;14(7):827. doi: 10.3390/biom14070827 (PMC11275138; doi:10.3390/biom14070827)

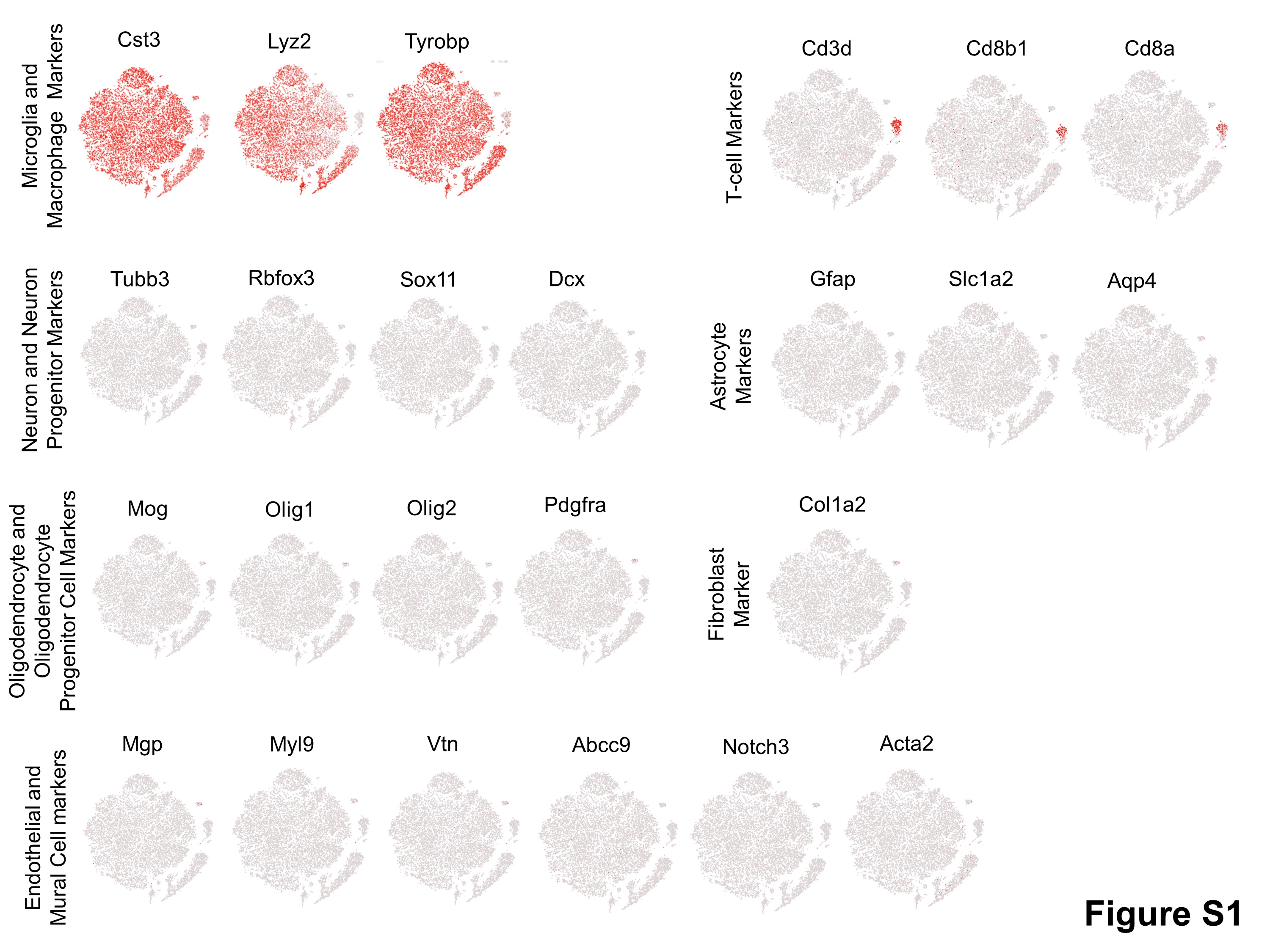

Supplement: Supplementary file 1 [file biomolecules-14-00827-s001.zip › Supplemental data/Figures/SuppFigure S1.jpg]

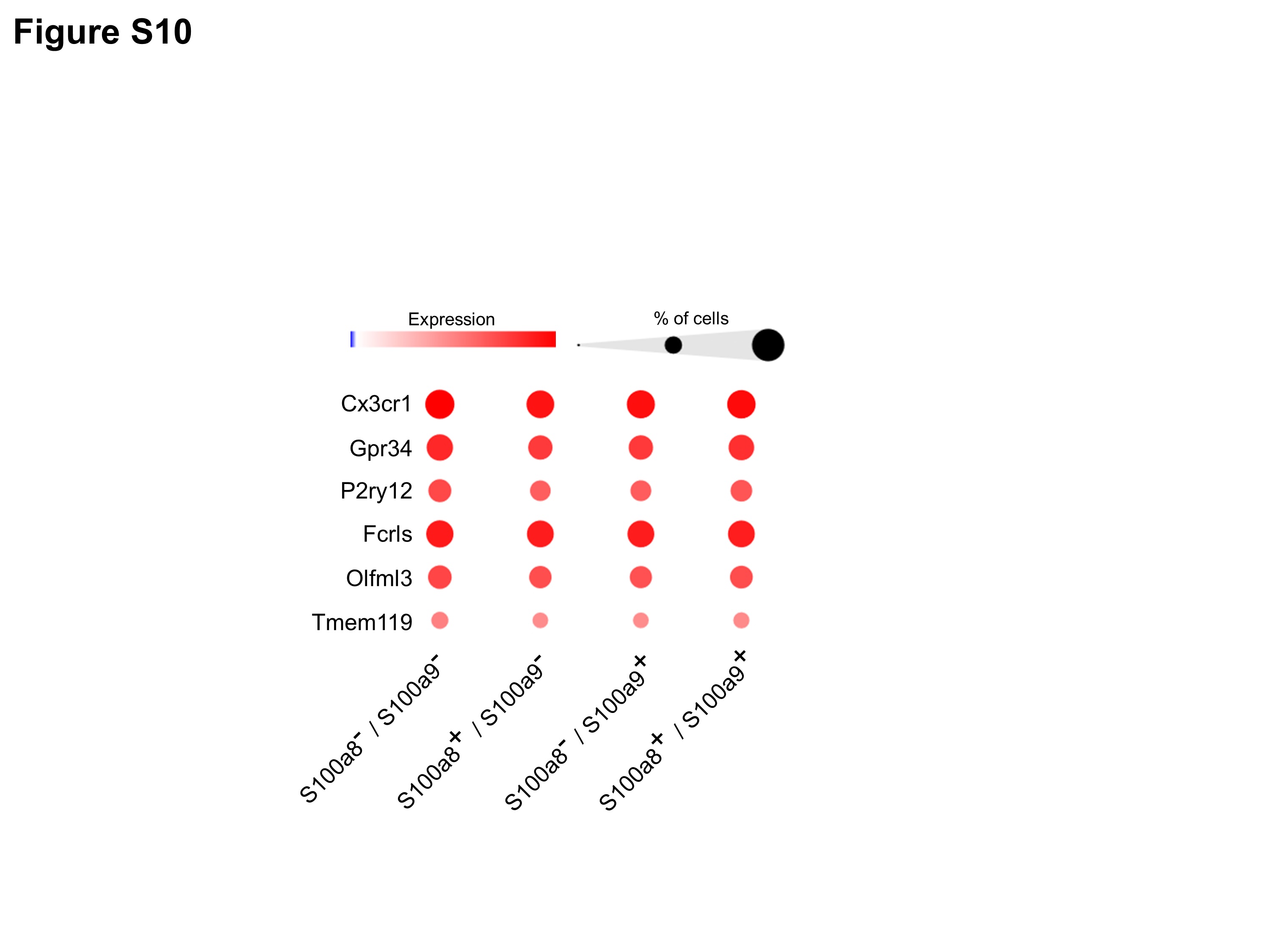

Supplement: Supplementary file 1 [file biomolecules-14-00827-s001.zip › Supplemental data/Figures/SuppFigure S10.jpg]

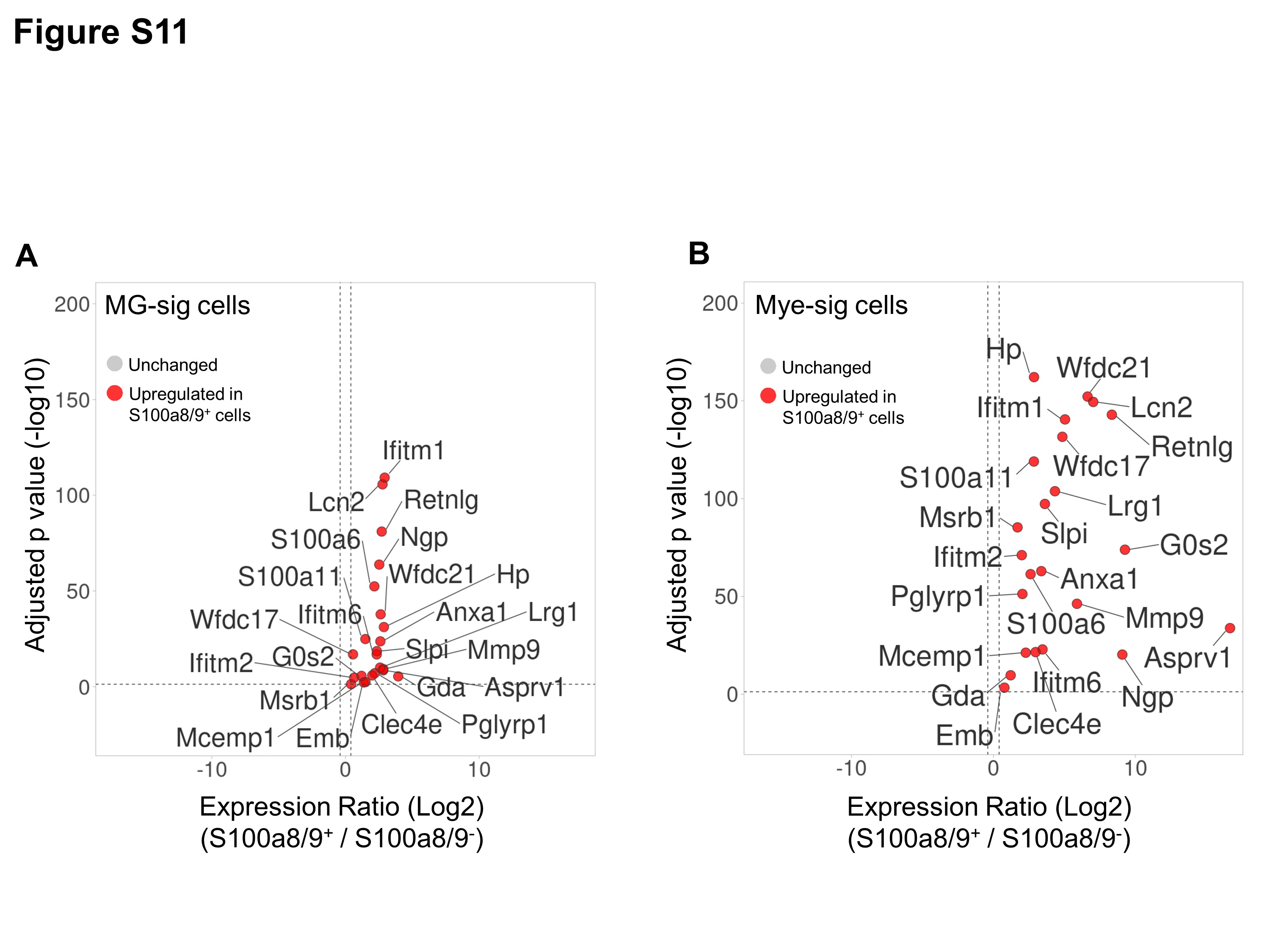

Supplement: Supplementary file 1 [file biomolecules-14-00827-s001.zip › Supplemental data/Figures/SuppFigure S11.jpg]

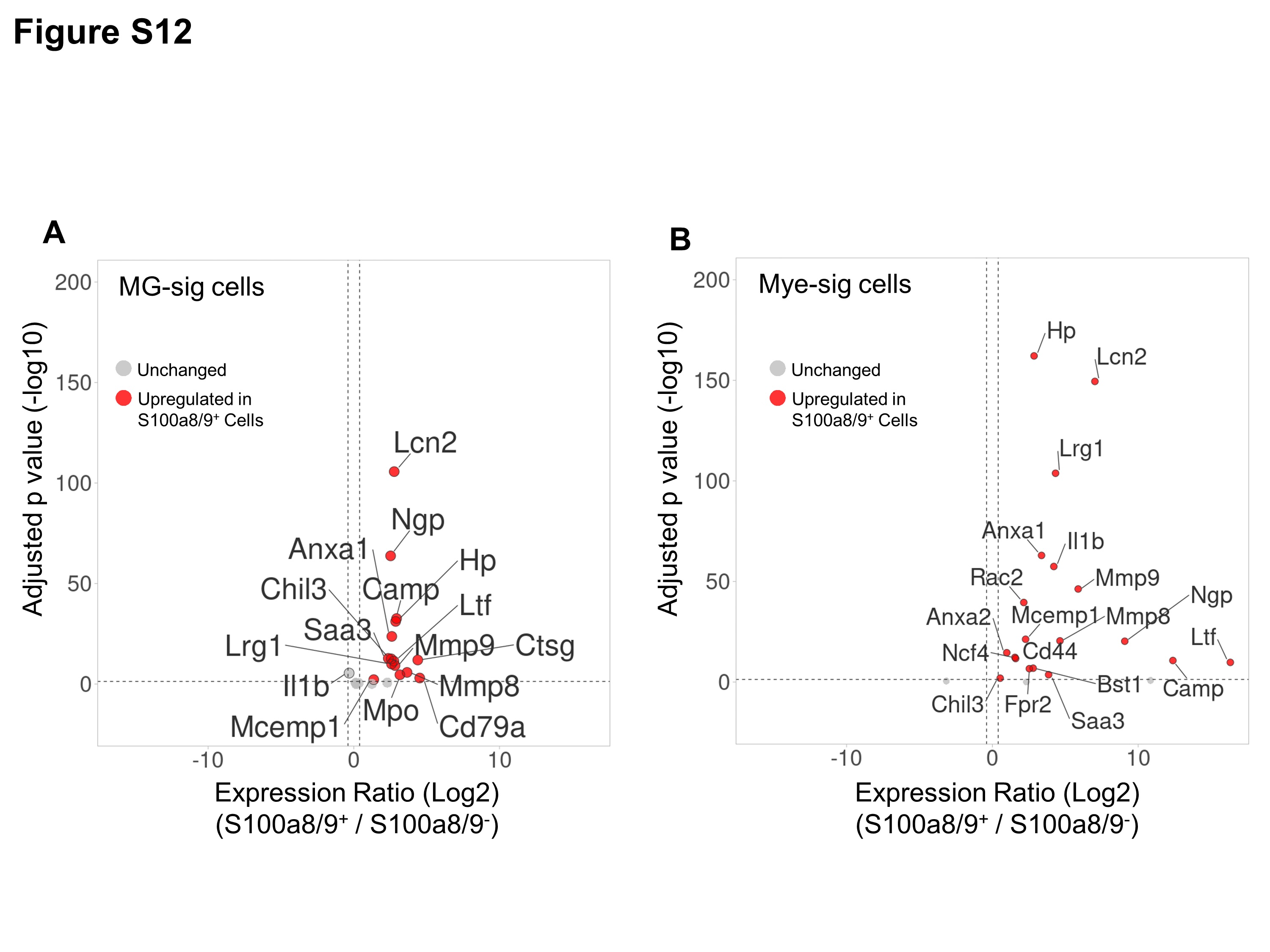

Supplement: Supplementary file 1 [file biomolecules-14-00827-s001.zip › Supplemental data/Figures/SuppFigure S12.jpg]

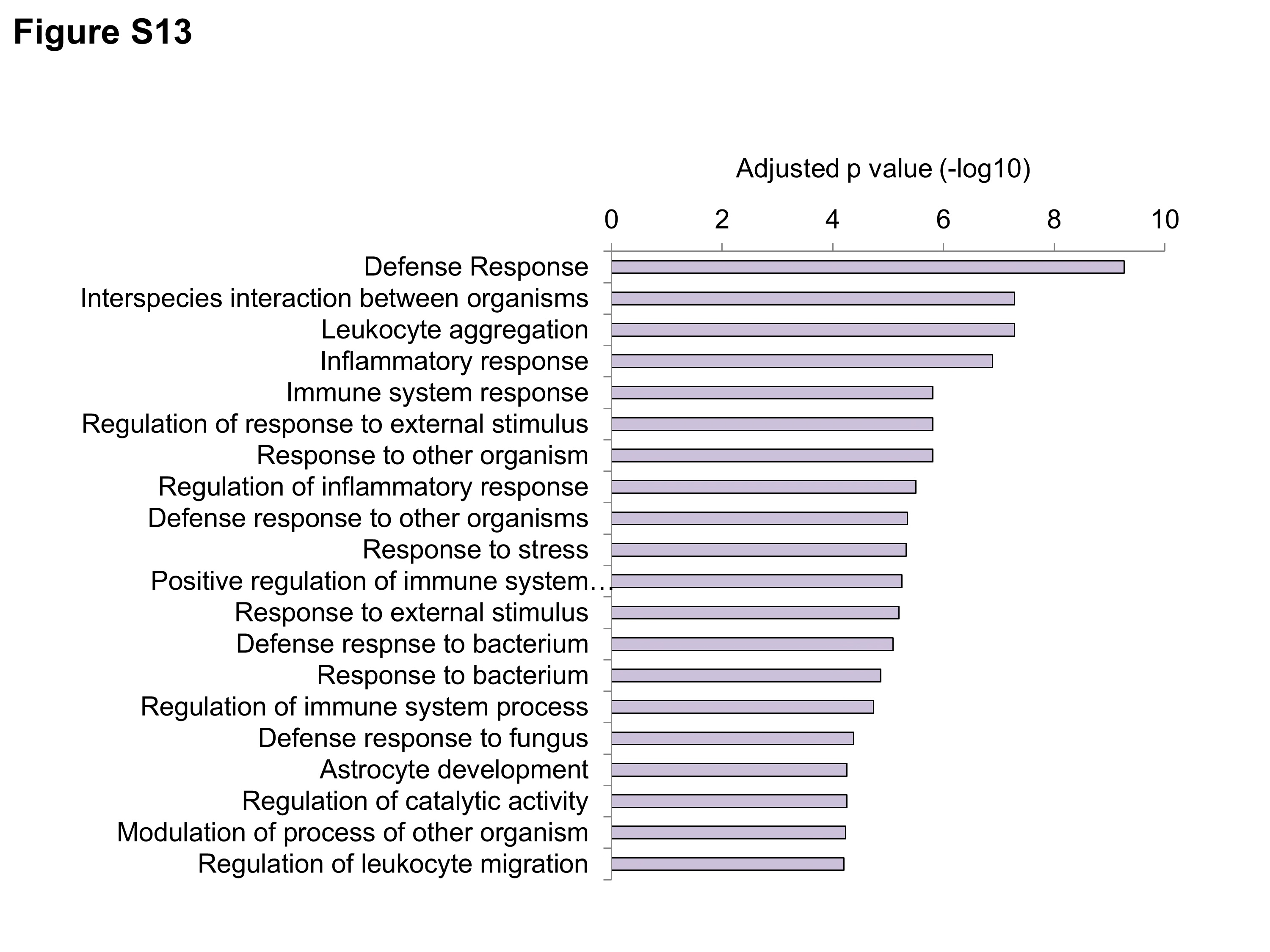

Supplement: Supplementary file 1 [file biomolecules-14-00827-s001.zip › Supplemental data/Figures/SuppFigure S13.jpg]

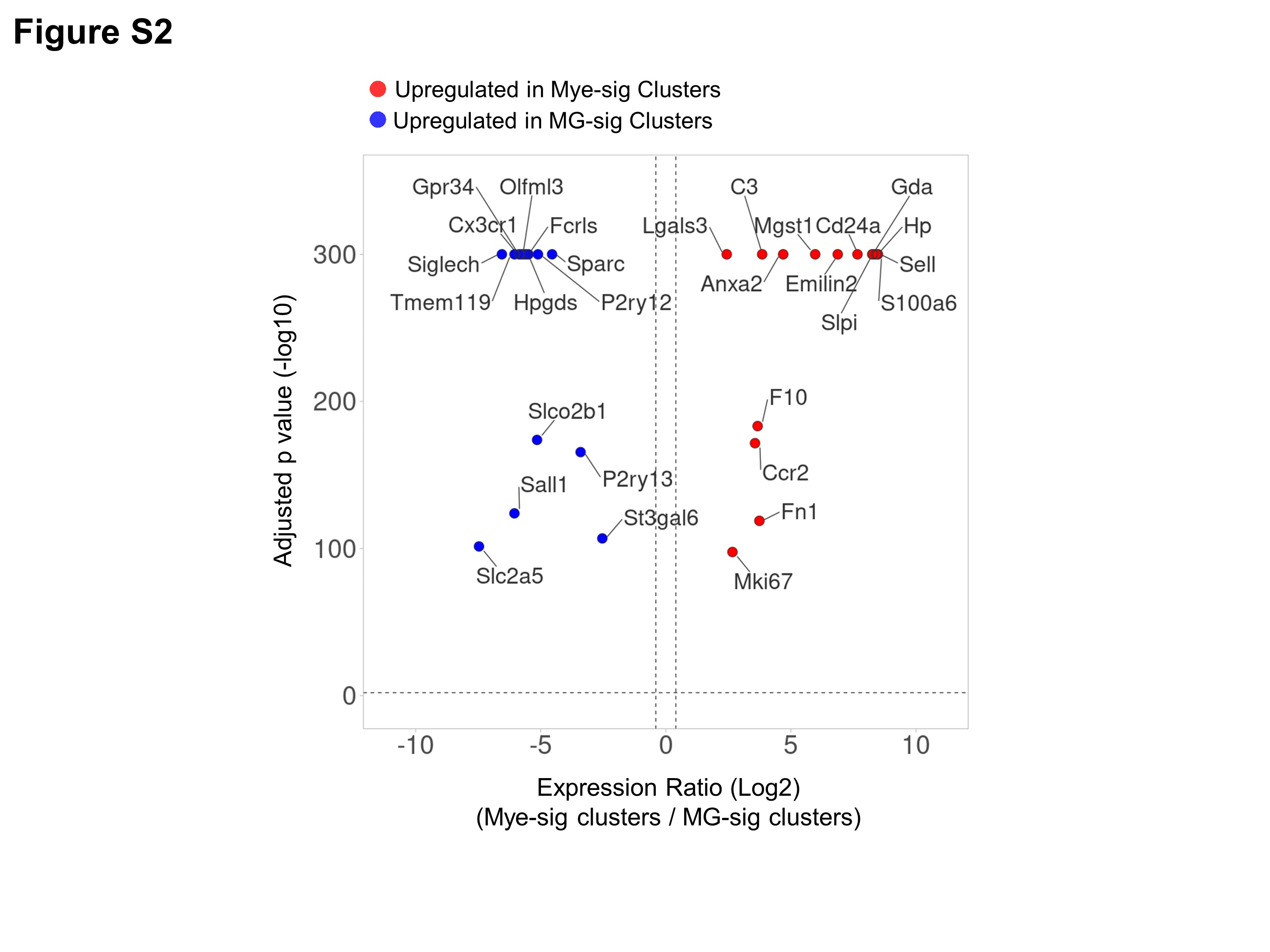

Supplement: Supplementary file 1 [file biomolecules-14-00827-s001.zip › Supplemental data/Figures/SuppFigure S2.jpg]

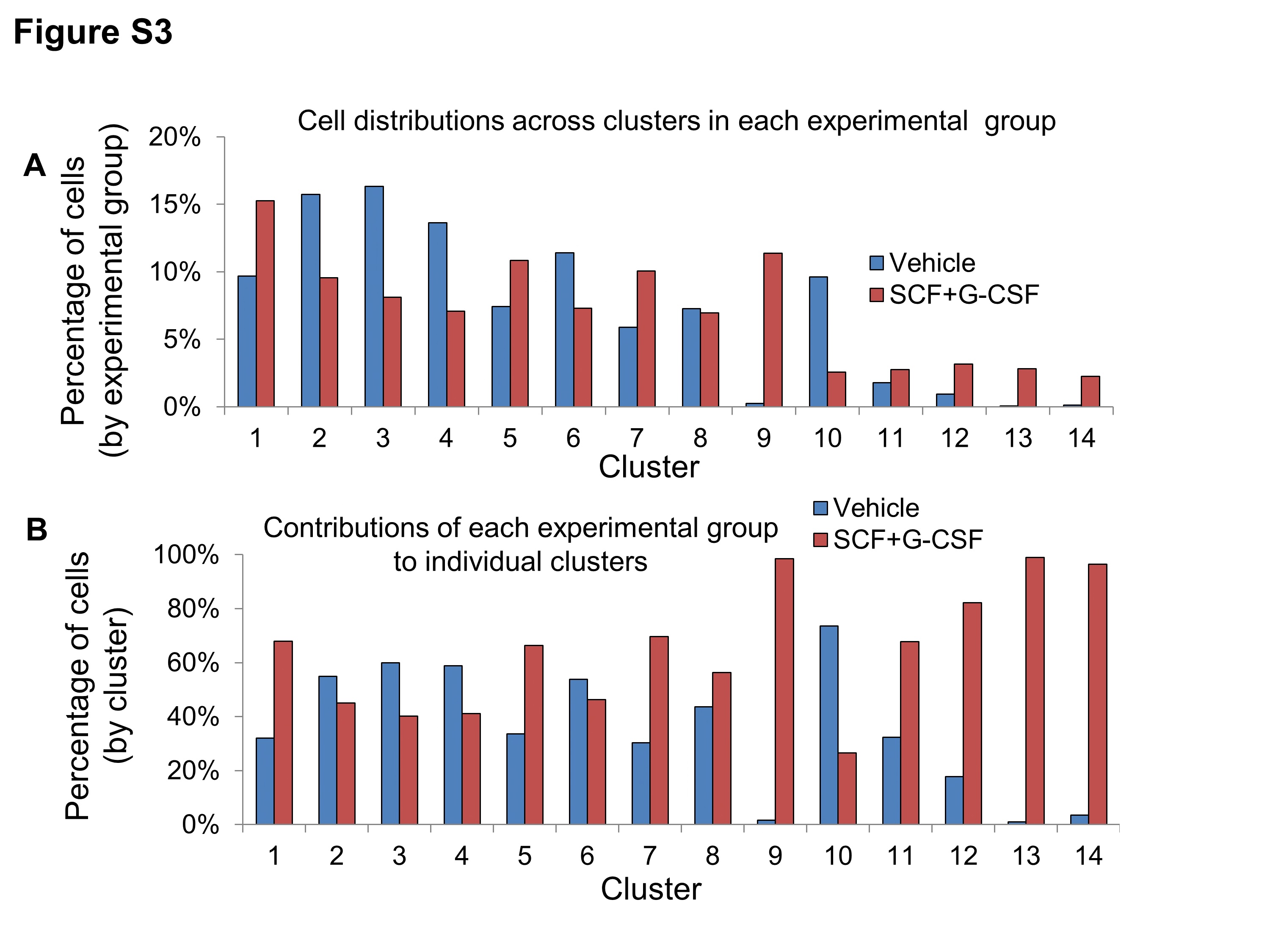

Supplement: Supplementary file 1 [file biomolecules-14-00827-s001.zip › Supplemental data/Figures/SuppFigure S3.jpg]

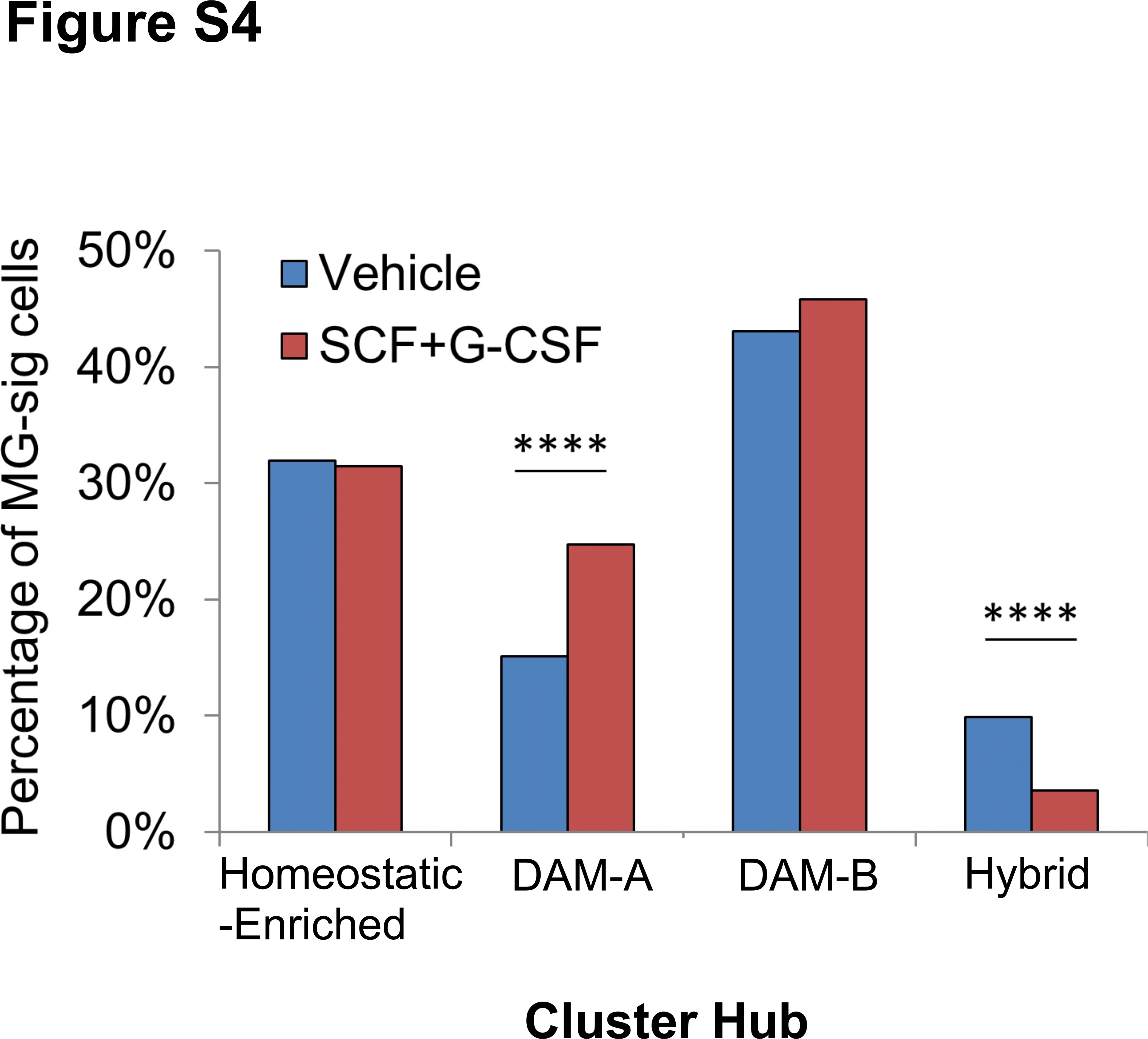

Supplement: Supplementary file 1 [file biomolecules-14-00827-s001.zip › Supplemental data/Figures/SuppFigure S4.jpg]

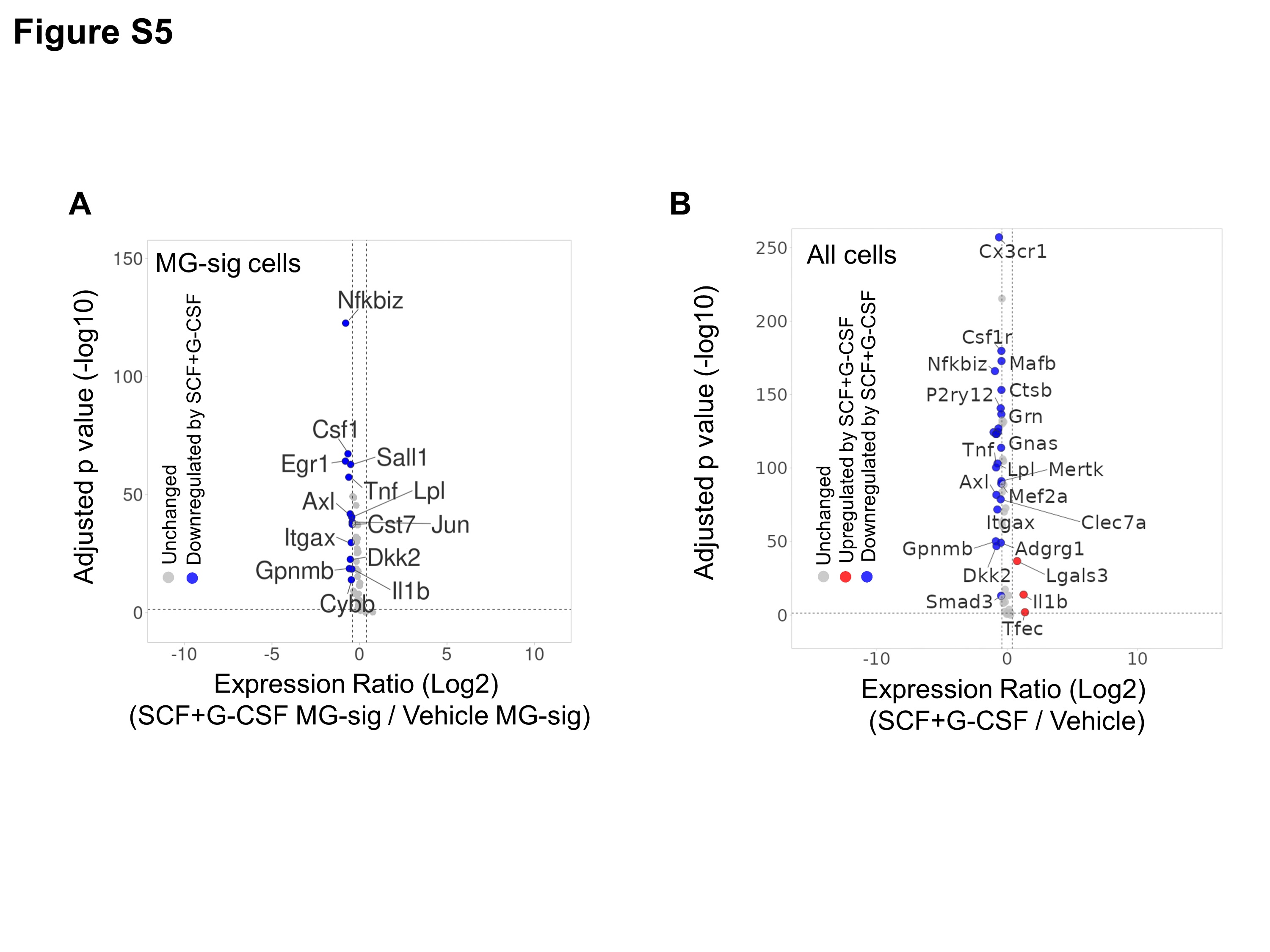

Supplement: Supplementary file 1 [file biomolecules-14-00827-s001.zip › Supplemental data/Figures/SuppFigure S5.jpg]

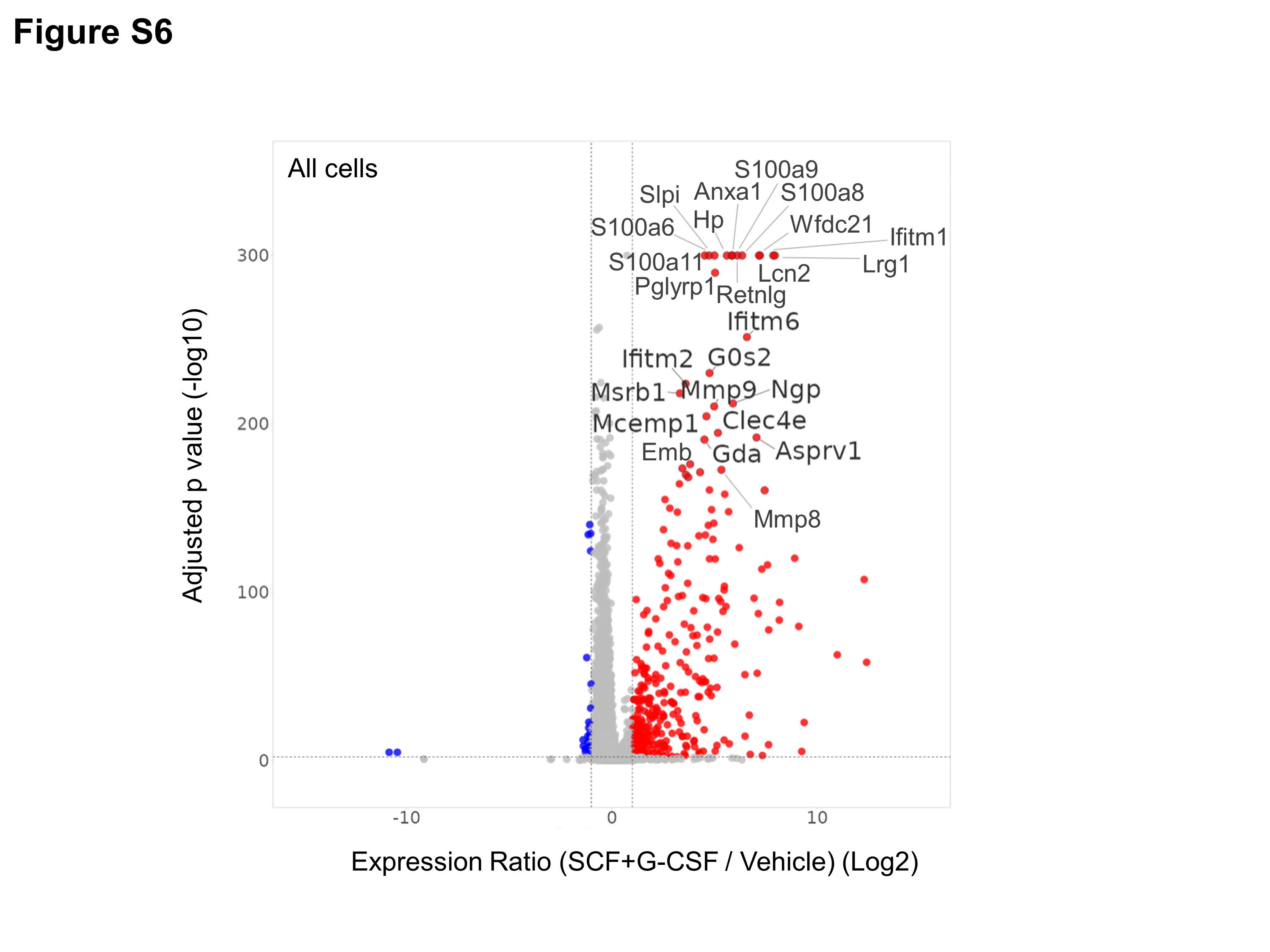

Supplement: Supplementary file 1 [file biomolecules-14-00827-s001.zip › Supplemental data/Figures/SuppFigure S6.jpg]

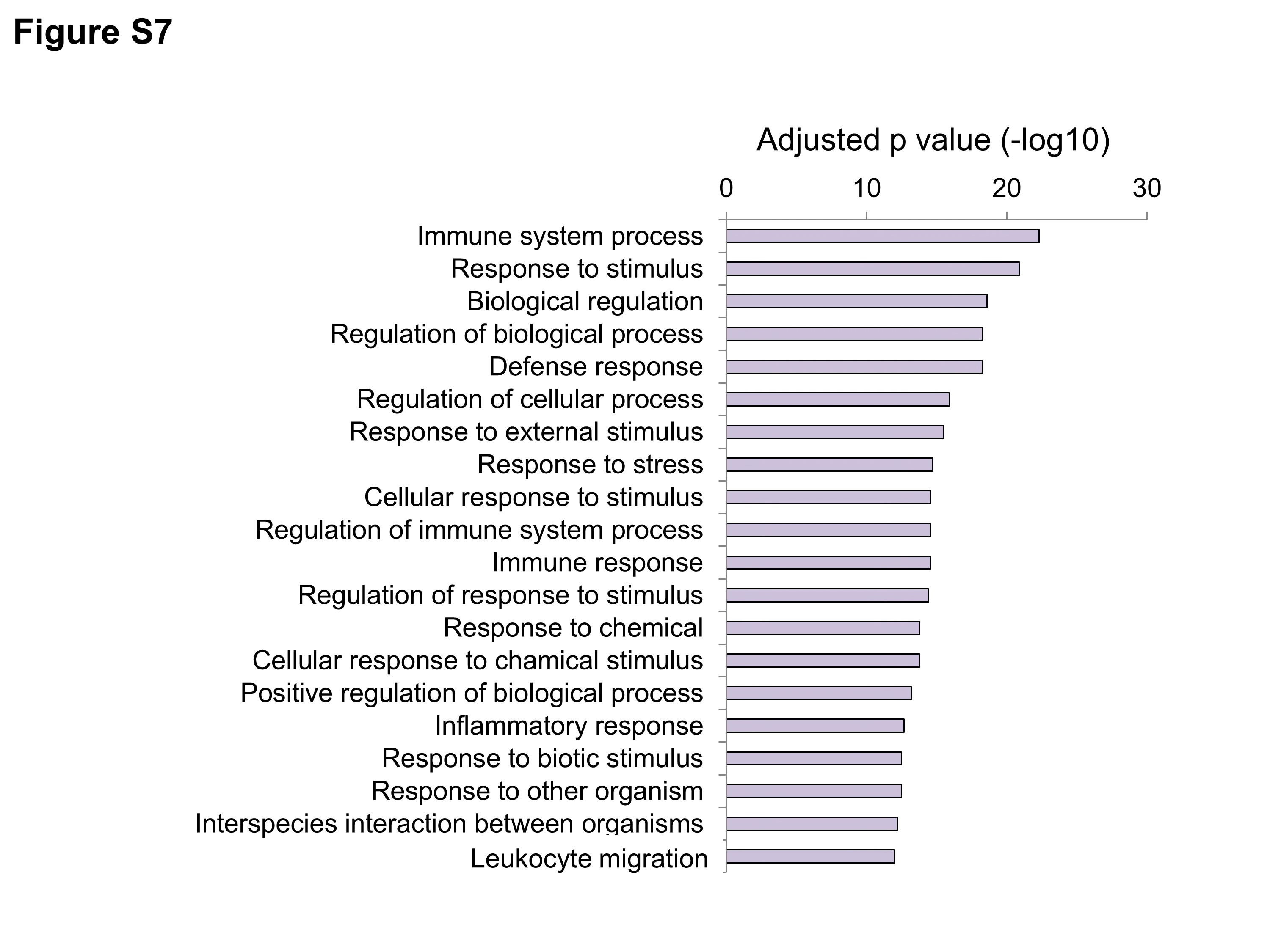

Supplement: Supplementary file 1 [file biomolecules-14-00827-s001.zip › Supplemental data/Figures/SuppFigure S7.jpg]

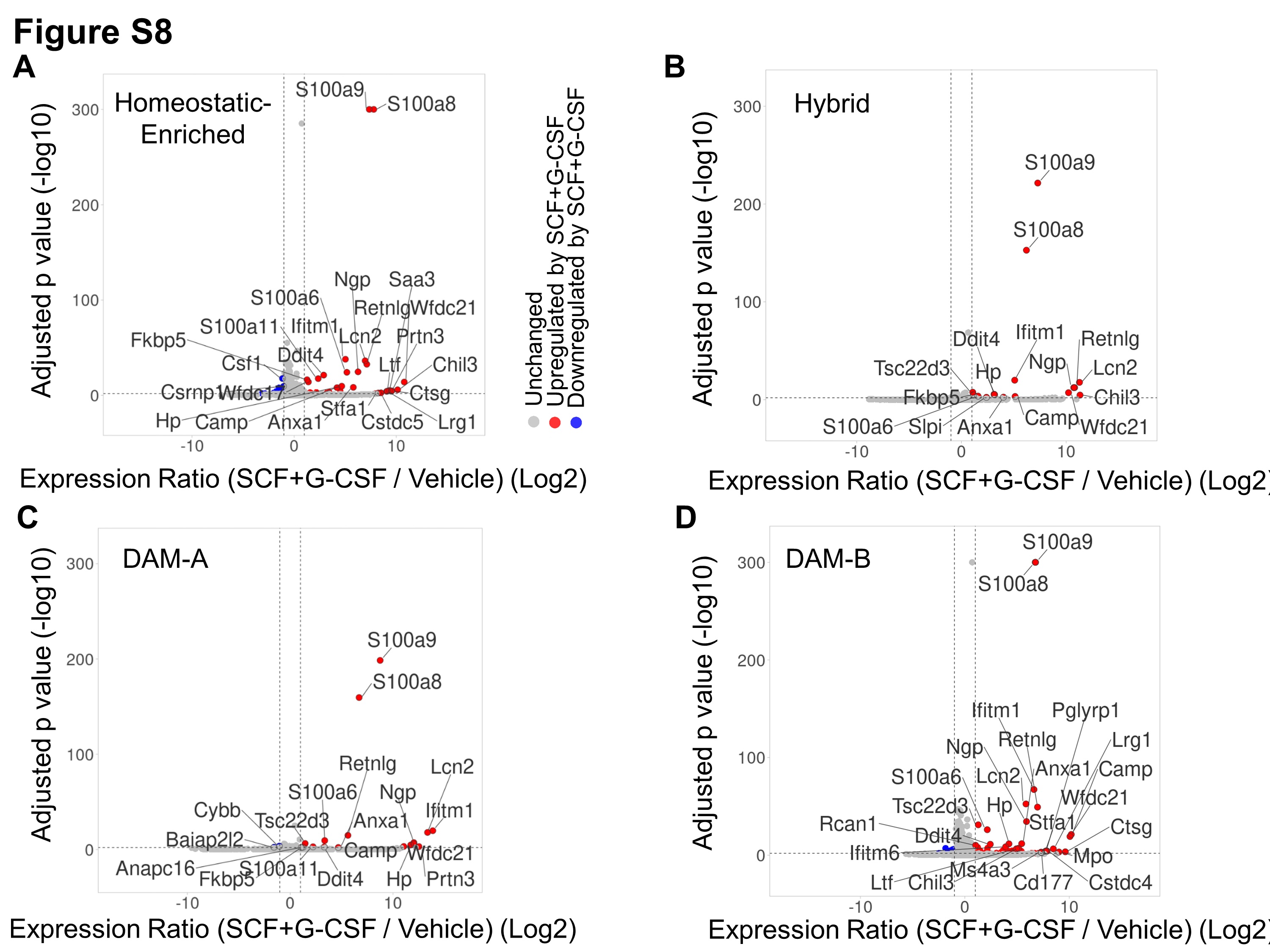

Supplement: Supplementary file 1 [file biomolecules-14-00827-s001.zip › Supplemental data/Figures/SuppFigure S8.jpg]

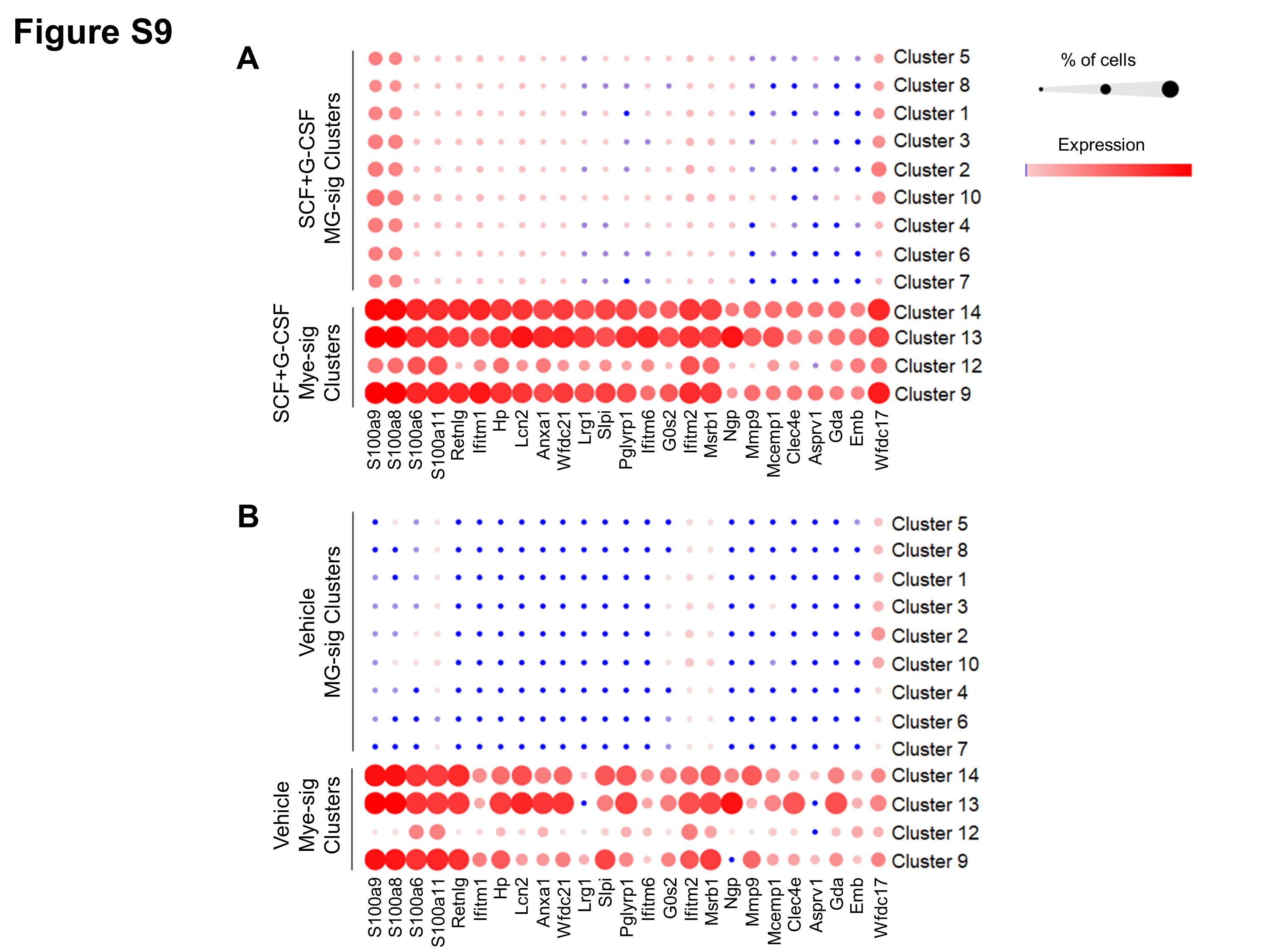

Supplement: Supplementary file 1 [file biomolecules-14-00827-s001.zip › Supplemental data/Figures/SuppFigure S9.jpg]

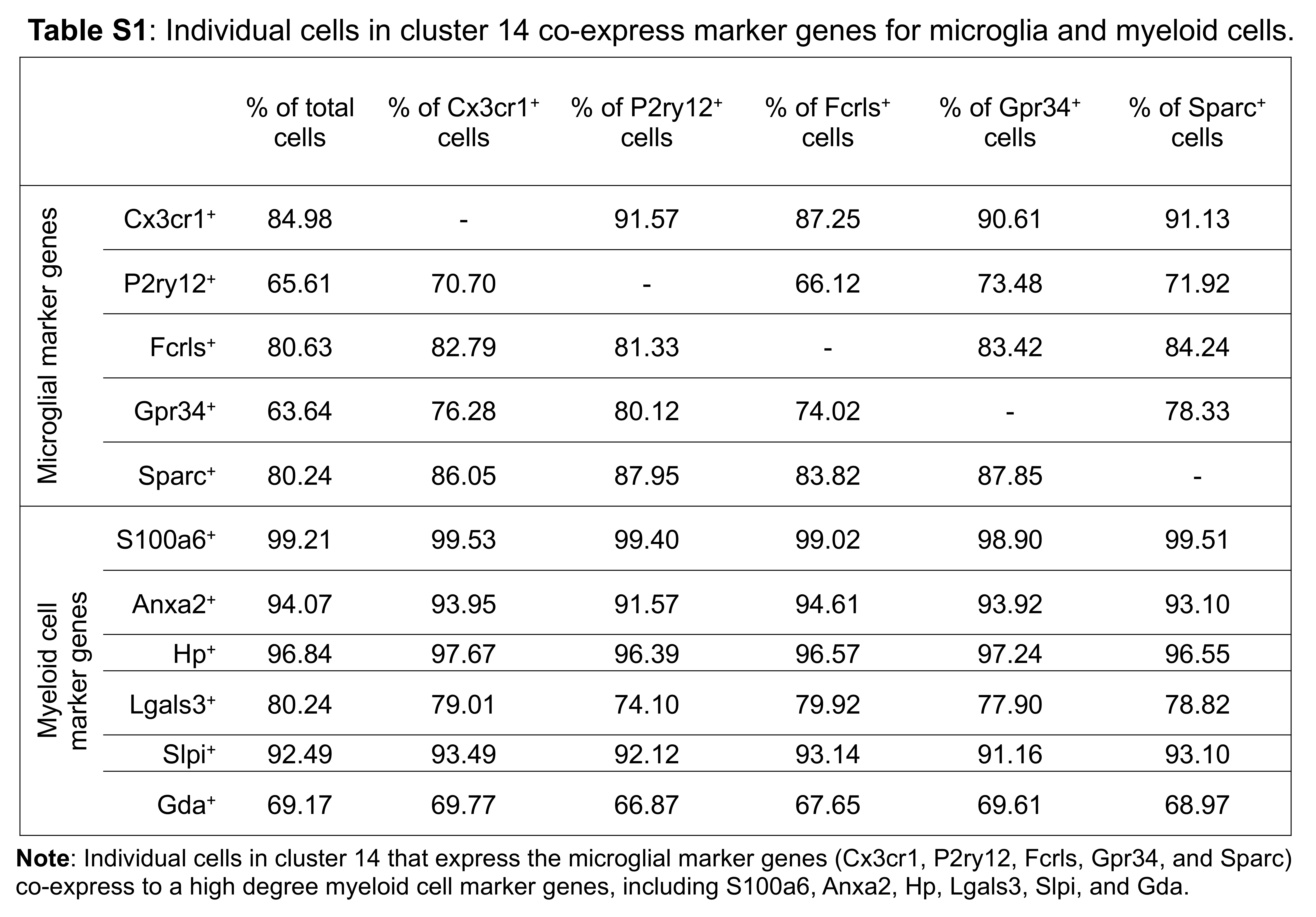

Supplement: Supplementary file 1 [file biomolecules-14-00827-s001.zip › Supplemental data/Tables/Table S1.jpg]

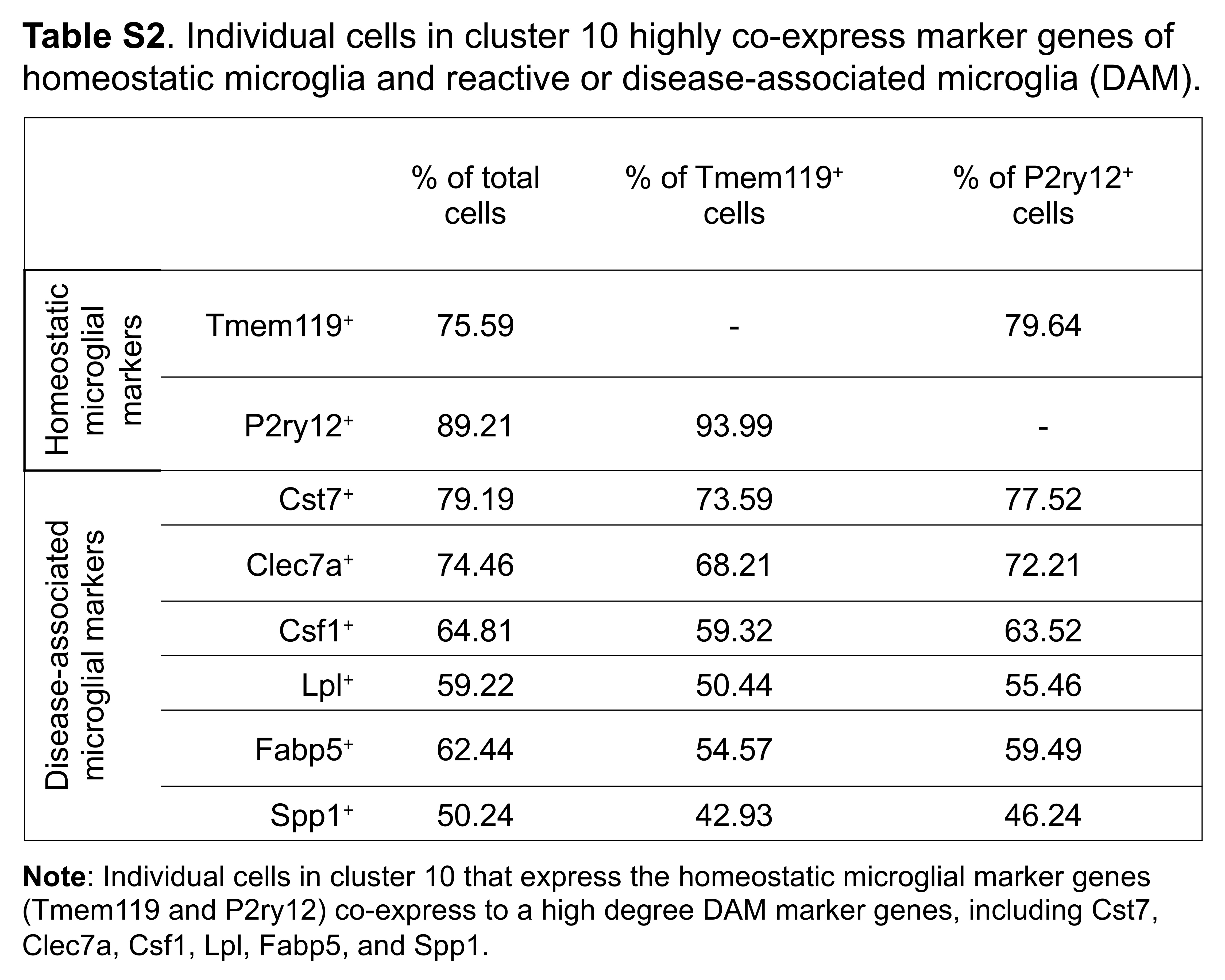

Supplement: Supplementary file 1 [file biomolecules-14-00827-s001.zip › Supplemental data/Tables/Table S2.jpg]
